# Tripeptide DT-109 (Gly-Gly-Leu) attenuates atherosclerosis and vascular calcification in nonhuman primates

**DOI:** 10.1038/s41392-025-02201-2

**Published:** 2025-04-07

**Authors:** Linying Jia, Pengxiang Qu, Yang Zhao, Liang Bai, Honghao Ren, Ao Cheng, Zeyao Ma, Cheng Ding, Yongjie Deng, Lingxuan Kong, Ying Zhao, Oren Rom, Yajie Chen, Naqash Alam, Wenbin Cao, Sixue Zhai, Zuowen Zheng, Zhi Hu, Lu Wang, Yabing Chen, Sihai Zhao, Jifeng Zhang, Jianglin Fan, Y. Eugene Chen, Enqi Liu

**Affiliations:** 1 Laboratory Animal Center, Xi’an Jiaotong University Health Science Center, Xi’an, Shaanxi 710061 China; 2 Department of Internal Medicine, University of Michigan Medical Center, 2800 Plymouth Road, Ann Arbor, MI 48109 USA; 3 Department of Biostatistics, School of Public Health, University of Michigan, 1415 Washington Heights, Ann Arbor, MI 48109 USA; 4 Department of Pathology and Translational Pathobiology, Department of Molecular and Cellular Physiology, Louisiana State University Health Sciences Center-Shreveport, Shreveport, LA 71103 USA; 5 Guangdong Province Key Laboratory, Southern China Institute of Large Animal Models for Biomedicine, School of Pharmacy and Food Engineering, Wuyi University, Jiangmen, Guangdong 529000 China; 6 Department of Imaging, the Second Affiliated Hospital of Xi’an Medical University, Xi’an, Shaanxi 710038 China; 7 Spring Biological Technology Development Co., Ltd, Fangchenggang, Guangxi 538000 China; 8 Department of Cardiovascular Medicine, the First Affiliated Hospital of Xi’an Jiaotong University, Xi’an, Shaanxi 710061 China; 9 Department of Pathology and Laboratory Medicine, Oregon Health & Science University; Research Department, Portland Veterans Affairs Medical Center, Portland, OR 97239 USA; 10 Cardiometabolic Innovation Center, Ministry of Education, Xi’an, Shaanxi 710061 China; 11 Institute of Cardiovascular Science, Translational Medicine Institute, Xi’an Jiaotong University Health Science Center, Xi’an, 710061 China

**Keywords:** Cardiology, Cardiovascular diseases

## Abstract

Advanced atherosclerotic lesions and vascular calcification substantially increase the risk of cardiovascular events. However, effective strategies for preventing or treating advanced atherosclerosis and calcification are currently lacking. This study investigated the efficacy of DT-109 (Gly-Gly-Leu) in attenuating atherosclerosis and calcification in nonhuman primates, exploring its broader therapeutic potential. In this study, twenty male cynomolgus monkeys were administered a cholesterol-rich diet *ad libitum* for 10 months. Then, the animals were treated either orally with DT-109 (150 mg/kg/day) or a vehicle (H_2_O) for 5 months while continuing on the same diet. Plasma lipid levels were measured monthly and at the end of the experiment, pathological examinations of the aortas and coronary arteries and RNA sequencing of the coronary arteries were performed. To explore possible molecular mechanisms, the effects of DT-109 on smooth muscle cells (SMCs) were examined in vitro. We found that DT-109 administration significantly suppressed atherosclerotic lesion formation in both the aorta and coronary arteries. Pathological examinations revealed that DT-109 treatment reduced lesional macrophage content and calcification. RNA sequencing analysis showed that DT-109 treatment significantly downregulated the pro-inflammatory factors *NLRP3*, *AIM2*, and *CASP1*, the oxidative stress factors *NCF2* and *NCF4*, and the osteogenic factors *RUNX2*, *COL1A1*, *MMP2*, and *MMP9*, while simultaneously upregulating the expression of the SMCs contraction markers *ACTA2*, *CNN1*, and *TAGLN*. Furthermore, DT-109 inhibited SMC calcification and NLRP3 inflammasome activation in vitro. These results demonstrate that DT-109 effectively suppresses both atherosclerosis and calcification. These findings, in conjunction with insights from our previous studies, position DT-109 as a novel multifaceted therapeutic agent for cardiovascular diseases.

## Introduction

Atherosclerosis and its complications, including myocardial infarction and stroke, are leading causes of death worldwide and remain a significant threat to human health. Atherosclerosis is caused by various genetic and environmental risk factors, such as hyperlipidemia, hypertension, diabetes mellitus, and cigarette smoking. In humans, it may take several decades for clinical complications to manifest.^1^ Atherosclerosis is widely recognized as a chronic vascular inflammation resulting from the interaction of these risk factors with arterial wall cells and atherogenic lipoproteins. Pathologically, it begins with the deposition of low-density lipoproteins (LDL), particularly oxidized LDL (ox-LDL). This is followed by the adhesion of circulating monocytes to the endothelial cells of the arteries and their migration into the subendothelial space, where they differentiate into macrophages. These macrophages then engulf the deposited ox-LDL through scavenger receptors, transforming it into foam cells that contribute to the formation of fatty streaks, a hallmark of early-stage atherosclerotic plaque.^2^ Over time, these fatty streaks progress into more advanced lesions characterized by the development of necrotic or lipid cores, rupture, hemorrhage, stenosis, as well as calcification, all of which can lead to severe cardiovascular and cerebrovascular events.^3^ Vascular calcification is a hallmark of advanced atherosclerosis, characterized by ectopic calcium deposition in arterial walls. This calcification can occur in both the intimal and medial layers of the arteries, contributing to increased plaque vulnerability (intimal calcification) and arterial stiffness (medial calcification) and the risk of cardiovascular events. Although the molecular mechanisms are not yet fully understood, calcification is now recognized as an active rather than passive and tightly regulated process, primarily driven by vascular smooth muscle cells (SMCs).^4–6^

It is widely believed that osteogenic phenotype changes in vascular SMCs resemble the process of bone formation, wherein these cells lose their contractility markers and undergo a phenotypic transition into chondrocytes, osteoblasts, and osteocytes.^7^ Despite this understanding, there are currently no effective therapeutics available for the prevention or treatment of vascular calcification in clinical practice.

Current clinical interventions for atherosclerotic diseases mainly focus on lipid-lowering medications, such as statins, PSCK9 inhibitors, and ezetimibe.^8^ However, many patients continue to face a high risk of cardiovascular events, even after effective lipid reduction, highlighting the need for the development of new therapies.^9,10^ Pharmacological approaches targeting calcification, including advanced glycation end-product crosslink breakers and angiotensin-converting enzyme inhibitors, are emerging as promising candidates to reduce arterial stiffness.^11–13^ The development of the new drugs depends significantly on animal models that closely replicate human disease.^14^ While genetically modified mice are the most commonly used animal models for studying atherosclerosis and lipid metabolism, significant disparities exist between these models and humans, which may prevent them from application in drug development. Notably, there are differences in the complexity and characteristics of atherosclerotic plaque pathology and lipid metabolism between species. For instance, the proportion of cholesterol carried by high-density lipoproteins (in mice) differs greatly from that carried by LDL (in humans), as does vascular pathology.^15,16^ Therefore, utilizing nonhuman primate models, which more accurately reflect human lipid metabolism and atherosclerosis, could greatly enhance the chances of successful clinical drug development.^17^

Recent reports have indicated that lower plasma glycine levels are clinically linked to various cardiovascular diseases.^18–21^ Glycine has demonstrated the ability to suppress ischemic injury in both the heart and brain, regulate glucolipid metabolism, and reduce blood pressure, while also significantly inhibiting calcium surges in lipopolysaccharide-induced macrophages.^22–24^ Additionally, we found that the glycine-based tripeptide DT-109 (Gly-Gly-Leu) effectively reduces atherosclerosis and metabolic dysfunction-associated steatohepatitis (MASH) in mice, primarily through the induction of glutathione (GSH) biosynthesis-mediated antioxidant effects, independent of any lipid-lowering mechanisms.^25,26^ We also reported that DT-109 treatment inhibited the progression of hepatic steatosis and fibrosis in nonhuman primates by modulating microbial bile acid metabolism.^27^ Based on these findings, we hypothesized that DT-109 may have beneficial effects on vascular pathology in nonhuman primates.

To investigate this hypothesis, we fed twenty male cynomolgus monkeys with metabolic disease a cholesterol-rich diet for 15 months. At 10 months, the monkeys were randomly divided into two groups of ten based on their nonalcoholic fatty liver disease activity score (NAS) and treated daily by oral gavage with either vehicle (H_2_O) or DT-109 (150 mg/kg/day) for the subsequent 5 months.^27^ The results indicated that DT-109 treatment significantly reduced the severity of aortic and coronary atherosclerosis, including reduced inflammatory cell infiltration and calcification in the cynomolgus monkeys. Our in vitro studies using cultured vascular SMC showed that the beneficial effects of DT-109 occur through multiple mechanisms, including the inhibition of inflammation, prevention of smooth muscle osteogenic phenotype switching, and reduction of oxidative stress. To the best of our knowledge, this is the first report demonstrating that tripeptide DT-109 can effectively reduce atherosclerosis in nonhuman primates, establishing it as a pioneering peptide-based therapeutic agent with significant potential in treating cardiovascular and metabolic diseases.

## Results

### DT-109 attenuates aortic and coronary atherosclerosis

**Fig. 1:**
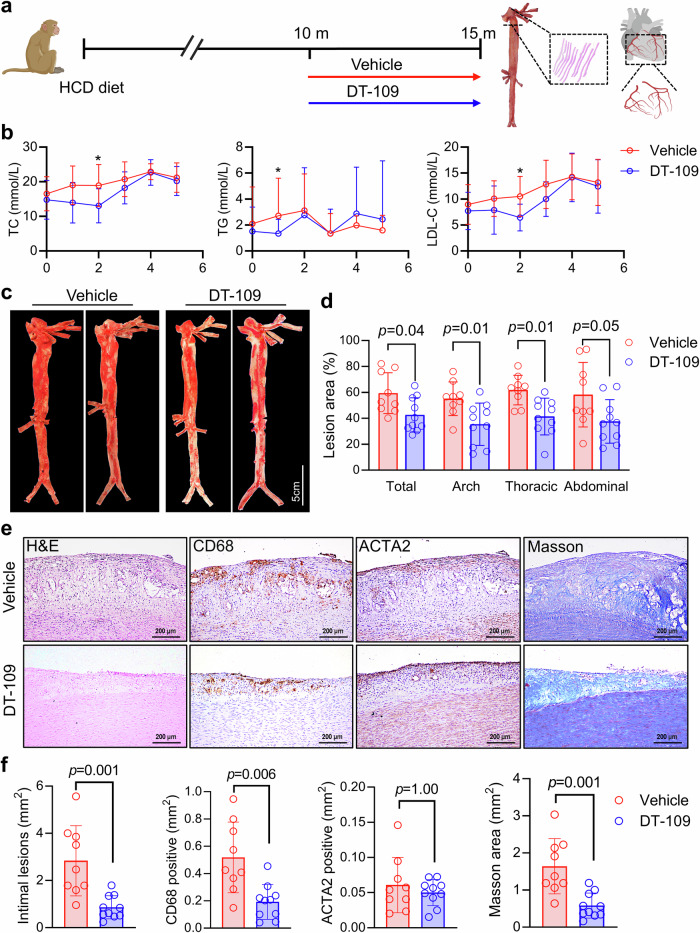
DT-109 ameliorates atherosclerotic lesions and macrophage infiltration. **a** Schematic diagram of the experimental approach. Twenty cynomolgus monkeys (male, age ≥ 9 years, BMI > 30) were fed a high cholesterol diet (HCD) for 10 months and then randomly divided to receive either DT-109 (150 mg/kg/d) or vehicle (H_2_O) for an additional 5 months, followed by pathological analysis of the aorta. **b** Lipid levels during 5 months of DT-109 treatment. Levels of total cholesterol (TC), triglycerides (TG), and low-density lipoprotein cholesterol (LDL-C) were measured (n = 10 for each group). Unpaired comparisons between vehicle and DT-109 groups at each time point were performed using the Kruskal-Wallis test; * p < 0.05. **c** *En face* monkey aorta staining with Sudan IV (vehicle, n = 9; DT-109, n = 10). **d** Statistical analysis of the aortic lesions and the percentage of plaque in each part of the aorta. **e** Histological staining of the aortic arch with hematoxylin and eosin (H&E), CD68 (macrophage marker), ACTA2 (actin alpha 2, smooth muscle cell marker, SMC), and Masson’s trichrome staining. Scale bar: 200 μm. **f** Statistical analysis of the aortic arch intimal lesions, CD68, ACTA2, and Masson staining areas (vehicle, n = 9; DT-109, n = 10). Data are presented as mean ± SD. All statistical comparisons between groups were evaluated by *Kruskal-Wallis* test. The exact *p* value is specified

To analyze the effect of DT-109 on the formation of complex plaques, we first developed an atherosclerosis model using cynomolgus monkeys. Twenty monkeys (male, age ≥ 9 years, body mass index (BMI) > 30) received a high cholesterol diet (HCD) for 10 months. Subsequently, they were randomized into either the DT-109 group treated with DT-109 (150 mg/kg/d) or the vehicle (H_2_O) group for 5 months (Fig. 1a) while remaining on the HCD. Plasma biochemical analyses did not show any significant effect on lipid levels after 5 months of DT-109 treatment, although total cholesterol (TC), triglycerides (TG), and LDL cholesterol (LDL-C) tended to reduce in the first 3 months (Fig. 1b). High-density lipoprotein cholesterol (HDL-C) levels were not significantly different between the two groups before and after DT-109 treatment (Supplementary Fig. 1a). Evaluating the impact of lipids requires examing both changes at individual time point and the overall change in hypercholesterolemia throughout the entire duration of the treatment. The overall change is typically evaluated using the area under the curve (AUC) of lipid levels over time. In this study, we calculated the AUC and found no significant difference in lipid levels between the two groups despite observing transient changes in lipid levels (Supplementary Fig. 1b). At the endpoint, the “aortic trees” were isolated for Sudan IV staining. The aortic arch was sectioned for histopathological analysis. Sudan IV staining revealed significantly less overall atherosclerotic lesions in the DT-109 treatment group (59.38 ± 15.80% in control group vs. 42.62 ± 13.21% in DT-109 group; *p* = 0.04). Regionally, DT-109 treatment showed less atherosclerotic lesions in the aortic arch (55.10 ± 12.96% in control group vs. 35.42 ± 16.31% in DT-109 group, *p* = 0.01), the thoracic aorta (61.79 ± 11.39% in control group vs. 41.38 ± 14.18% in DT-109 group; *p* = 0.01). However, the difference was insignificant in the abdominal aorta (58.19 ± 24.92% in control group vs. 37.62 ± 16.77% in DT-109 group; *p* = 0.05) (Fig. 1c, d). We then performed histopathological analysis of the aortic arch lesions. In monkeys fed an HCD, atherosclerotic lesions are characterized by having fatty streaks, fibrous plaques, and advanced lesions similar to those of human atherosclerosis (Supplementary Fig. 1c, d). Furthermore, some advanced lesions showed a fibrous cap and a large lipid core in which cholesterol crystals and calcification were frequently present (Supplementary Fig. 1d, e), indicating that this monkey model can simulate the pathology of human atherosclerosis. Regardless of the lesion features, the whole aortic arch of each animal was serially sectioned for the analysis of the microscopic size, cellular components, and extracellular matrix. DT-109 treatment resulted in a significant decrease in the intimal lesion (2.84 ± 1.49 mm^2^ in control group vs. 0.86 ± 0.49 mm^2^ in DT-109 group; *p* = 0.001) accompanied by reduction of lesional macrophages (0.52 ± 0.26 mm^2^ in control group vs. 0.19 ± 0.13 mm^2^ in DT-109 group; *p* = 0.006), and reduction in the collagen content (1.64 ± 0.74 mm^2^ in control group vs. 0.58 ± 0.34 mm^2^ in DT-109 group; *p* = 0.001) while no difference in the SMCs compared to the vehicle (0.06 ± 0.04 mm^2^ in control group vs. 0.05 ± 0.012mm^2^ in DT-109 group; *p* = 1.00) (Fig. 1e, f). These results suggest that DT-109 ameliorates atherosclerosis by reducing lesional macrophages.

**Fig. 2:**
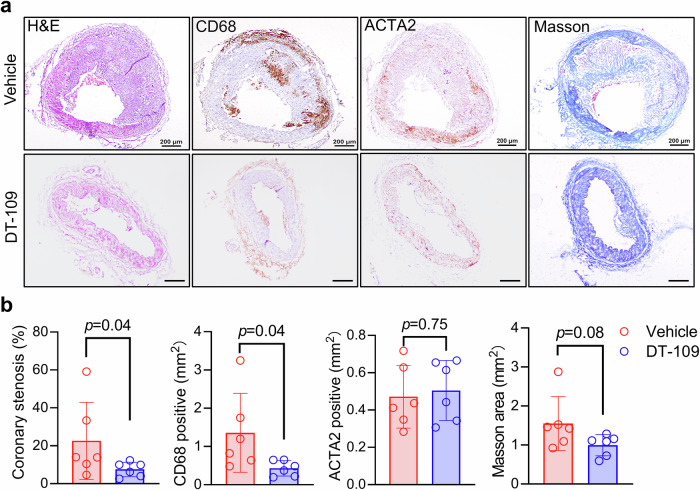
DT-109 treatment alleviates atherosclerosis in the coronary arteries. **a** Representative images of circumflex artery H&E staining, CD68 (for macrophage, MΦ), ACTA2 (for SMC), and Masson’s trichrome staining (vehicle, *n* = 6; DT-109, *n* = 6). Scale bar: 200 μm. **b** Statistical analysis of coronary stenosis rates and MΦ, SMC, Masson’s trichrome staining positive areas. Data are presented as mean ± SD. All statistical comparisons between groups were evaluated by *Kruskal-Wallis* test. The exact *p* value is specified

The development of coronary atherosclerosis can result in angina pectoris, myocardial infarction, and sudden death.^28^ However, the unique morphology and location of coronary arteries in humans present significant challenges for coronary atherosclerosis studies in small animal models.^29^ We isolated the right coronary artery, the anterior descending branch of the left coronary artery along with the circumflex from the monkeys (Supplementary Fig. 2a). The right coronary artery was used for RNA sequencing, whereas the corresponding left coronary artery was used for the pathological analysis of atherosclerotic lesions. There was no difference in the heart to body weight ratio between the DT-109-treated and vehicle groups (Supplementary Fig. 2b). Pathological analysis of the left coronary circumflex showed DT-109 treatment caused a significant reduction in coronary stenosis (22.56 ± 20.35% vs. 7.5 ± 3.82%; *p* = 0.04) and macrophage infiltration (1.36 ± 1.03 mm^2^ vs. 0.43 ± 0.20 mm^2^; *p* = 0.04), but not that for SMCs (0.47 ± 0.17 mm^2^ vs. 0.50 ± 0.16 mm^2^; *p* = 0.75) or collagen content (1.55 ± 0.69 mm^2^ vs. 0.99 ± 0.27 mm^2^; *p* = 0.08) (Fig. 2). These results demonstrated that DT-109 significantly inhibited coronary atherosclerosis in addition to aortic lesions.

### DT-109 attenuates vascular calcification in nonhuman primates

**Fig. 3:**
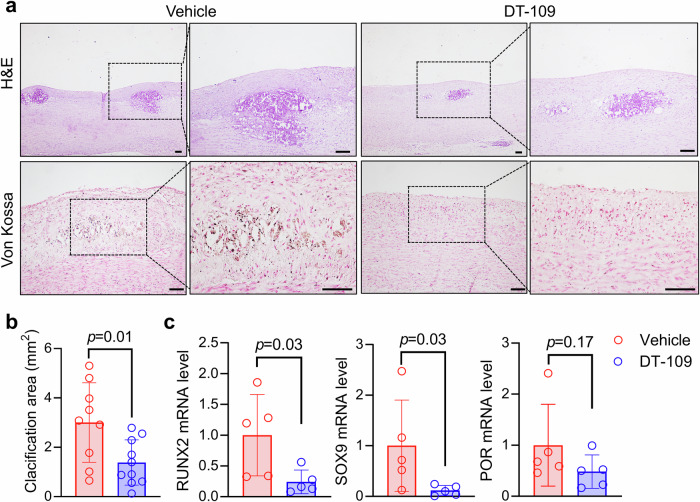
DT-109 attenuates vascular calcification in cynomolgus monkeys. **a** Hematoxylin and eosin (H&E) and calcification staining (Von Kossa) of the aortic arch (scale bar: 100 μm). **b** Quantification of calcified area in the aortic arch (vehicle, *n* = 9; DT-109, *n* = 10). **c** Extraction of total RNA from monkey common carotid arterial tissue and detection of runx family transcription factor 2 (*RUNX2*), SRY-box transcription factor 9 (*SOX9)*, cytochrome p450 oxidoreductase (*POR*) mRNA expression by real-time PCR (*n* = 5 for each group). Data are presented as mean ± SD. All statistical comparisons between groups were evaluated by *Kruskal–Wallis* test

As described above, atherosclerotic lesions were often associated with calcification. Calcification in the lesions can be observed under light microscopy using hematoxylin and eosin (H&E)-stained specimens. Calcification exhibits diverse microscopic features, including the deposition of calcium materials or vesicles embedded in the extracellular matrix either focally or diffusely and patches of calcium sizing over 200 μm (Supplementary Fig. 3a–c). Moreover, calcification can be easily observed by von Kossa staining. We found that DT-109 treatment significantly reduced the calcified area of the aortic arch (3.00 ± 1.61 mm^2^ vs. 1.38 ± 0.92 mm^2^; *p* = 0.009) (Fig. 3a, b). Furthermore, we performed micro-CT scans of the thoracic aorta and statistically analyzed the area of the scanned calcification foci. The results indicated that the calcified lesions in the thoracic aorta were also significantly reduced after DT-109 administration (4.78 ± 1.74 mm^2^ vs. 1.81 ± 1.26 mm^2^; *p* = 0.02) (Supplementary Fig. 4a). To elucidate the possible molecular mechanisms involved in the inhibitory effect of DT-109 on calcification, we measured the protein levels of genes related to vascular calcification in monkeys and showed that DT-109 treatment reduced the protein levels of RUNX family transcription factor 2 (RUNX2) and SRY-box transcription factor 9 (SOX9) (Supplementary Fig. 4c). Similarly, we examined the expression of genes related to calcification in monkey common carotid arteries, including, *RUNX2*, *SOX9*, cytochrome p450 oxidoreductase (*POR*), and bone morphogenetic protein 2 (*BMP2*). Real-time PCR results showed that the osteogenic transcription factors *RUNX2* and *SOX9* were downregulated after DT-109 administration in the common carotid arteries (Fig. 3c and Supplementary Fig. 4b).

### Transcriptome analysis reveals DT-109-mediated inhibition of inflammation and oxidative stress

**Fig. 4:**
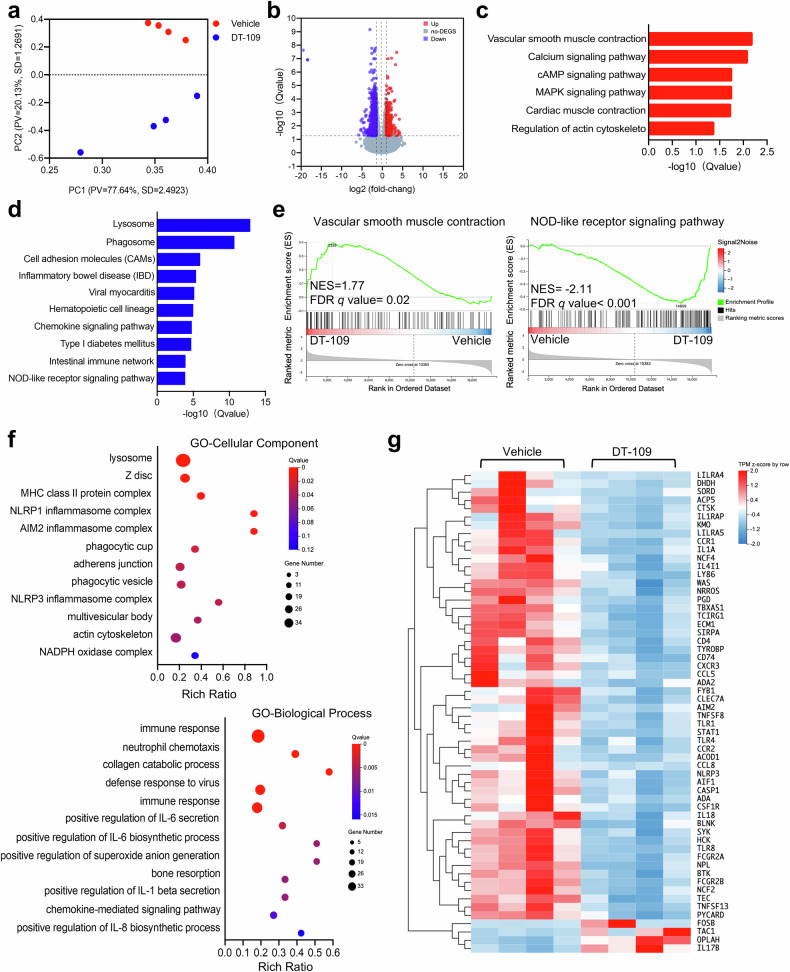
Transcriptomics reveals that DT-109 induces vascular smooth muscle contraction and inhibits inflammation.RNA-sequencing was performed on the right coronary artery from cynomolgus monkeys after treatment with vehicle or DT-109 for 5 months (*n* = 4 for each group). **a** Principal components analysis (PCA) of RNA-sequencing data. **b** Volcano plots of differentially expressed genes (DEGs) (blue: down-regulated; red: upregulated. log2 fold change ≥ 1.5, Q value ≤ 0.05). **c, d** The Kyoto Encyclopedia of Genes and Genomes (KEGG) pathway was enriched for DEGs up-regulated and down-regulated in red and blue, respectively. **e** Gene set enrichment analysis (GSEA) shows enrichment in the vascular smooth muscle contraction and NOD-like receptor signaling pathway. **f** Gene ontology (GO) enrichment analysis for DEGs, focusing on cellular components (GO-CC) and biological processes (GO-BP). **g** Heat map of coronary-related DEGs involved in inflammatory and oxidative stress responses

RNA-sequencing of the right coronary artery was performed to elucidate potential mechanisms by which DT-109 ameliorates atherosclerosis. Principal component analysis (PCA) revealed a significant separation between the treatment groups (Fig. 4a), with 600 differentially expressed genes (DEGs) significantly downregulated and 331 DEGs significantly upregulated by DT-109 (log2 fold change value ≥ 1.5, Q value < 0.05, Fig. 4b). Subsequent pathway enrichment analysis showed that DT-109 treatment significantly upregulated vascular smooth muscle contraction in the coronary arteries (Fig. 4c), while lysosome, cell adhesion molecules, type I diabetes mellitus, NOD-like receptor signaling pathway were downregulated (Fig. 4d). Similarly, gene set enrichment analysis (GSEA) showed a significant upregulation of vascular smooth muscle contraction and a significant downregulation of the NOD-like receptor signaling pathway after DT-109 treatment (Fig. 4e). Finally, gene ontology (GO) analysis indicated a significant enrichment of DEGs in the NLR family pyrin domain containing 1 (*NLRP1*), absent in melanoma 2 (*AIM2*), and NLR family pyrin domain containing 3 (*NLRP3*) inflammasome complexes, and NADPH oxidase complex within the cellular component (GO-cellular component), a significant enrichment of DEGs in the inflammatory response and inflammation-related pathways within the biological process component (GO-biological process), and an enrichment of DEGs in the toll-like receptor 4 binding, arachidonic acid binding, and 2-alkenal reductase [NAD(P)] activity within the molecular function component (GO-molecular function) (Fig. 4f and Supplementary Fig. 5). Heatmap analysis of coronary DEGs further highlighted essential genes regulating inflammatory responses (e.g., *NLRP3*, *AIM2*, *ASC*, *CASP1*, and *IL-18*) and oxidative stress (e.g., *NCF2* and *NCF4*) are significantly downregulated after DT-109 treatment; further demonstrating the substantial role of DT-109 in regulating inflammation and oxidative stress in coronary artery (Fig. 4g).

In addition, to assess the similarity between the nonhuman primate model and human atherosclerosis, we compared the DEGs from the monkey model with data from 29 human samples, including early and advanced atheromatous plaques.^30^ The results showed a very high consistency of changes in differential gene pathway enrichment in cynomolgus monkeys and human plaques. Notably, critical pathways affecting atherosclerosis, such as inflammatory and oxidative response-related pathways, were significantly downregulated in humans and monkeys (e.g., NOD-like receptor signaling pathway, Toll-like receptor signaling pathway, and oxidative phosphorylation) (Supplementary Fig. 6). Thus, unbiased transcriptomics showed that DT-109 induces vascular smooth muscle contraction and inhibits activation of coronary atherosclerotic inflammation and oxidative stress responses in nonhuman primates.

### DT-109 reduces inflammation and oxidative stress in cultured SMC

**Fig. 5:**
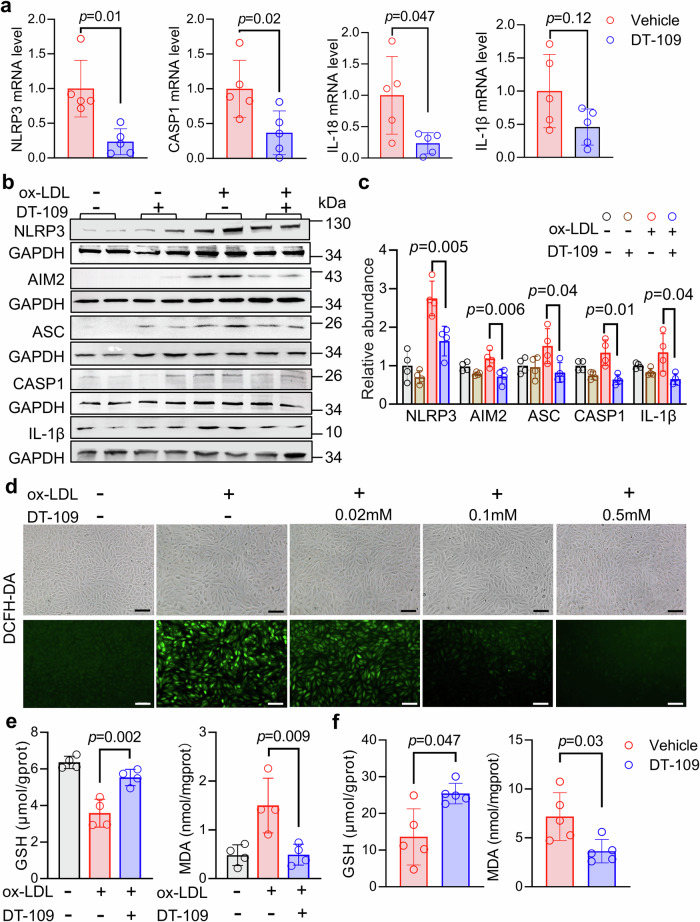
DT-109 inhibits inflammation and oxidative stress.**a** Coronary arteries were collected and analyzed for inflammation-associated genes. Real-time PCR analysis of NLR family pyrin domain containing 3 (*NLRP3*), apoptosis-related cysteine peptidase (*CASP1*), interleukin 18 (*IL-18*), and *IL-1β* expressions in the vehicle and DT-109 treated coronary arteries (normalized to 18S; *n* = 5 for each group). **b**, **c** Upon 24 h of ox-LDL induction, protein levels of NLRP3, absent in melanoma 2 (AIM2), PYD and CARD domain containing (ASC), CASP1, and IL-1β were determined by Western blot in A7r5 cells after vehicle or DT-109 treatment (*n* = 4 for each group). **d** A7r5 cells were divided into a control group and an ox-LDL-induced group, treated with or without DT-109 (0.02 mM, 0.1 mM, 0.5 mM), and labeled with DCFH-DA to assess superoxide levels (scale bar: 200 μm). **e** After ox-LDL stimulation, supernatants from A7r5 cells incubated with or without DT-109 (0.5 mM) were collected to analyze glutathione (GSH) and malondialdehyde (MDA) levels (*n* = 4 for each group). **f** Detection of GSH and MDA in carotid artery tissue (*n* = 5 for each group). Data are presented as mean ± SD. Statistical differences were compared using one-way ANOVA with *Tukey’s post hoc* analysis or *Dunn’s* test, multiple comparison adjusted *p*-values were reported

We first examined the effects of DT-109 on ox-LDL-induced SMC migration, contraction, and proliferation, and results showed that DT-109 had no significant effect on these processes (Supplementary Fig. 7a–g). Then, we assessed key genes in the relevant pathways at the RNA and protein levels to verify if the transcriptional changes we observed translated to functional outcomes. We performed real-time PCR validation using coronary tissue from monkeys and confirmed that DT-109 down-regulated *NLRP3, CASP1*, and *IL-18* expression (Fig. 5a). Additionally, DT-109 treatment reduced the levels of the protein NLRP3, AIM2, ASC, CASP1, and IL-1β in cultured rat aortic SMC line, A7r5 (Fig. 5b, c). Similar results were observed in human aortic smooth muscle cells (HASMCs) (Supplementary Fig. 8a). Subsequently, we differentiated THP-1 monocytes into macrophages using phorbol 12-myristate 13-acetate (PMA) to test whether DT-109 similarly affects the expression of inflammation-related genes in macrophages. The results showed that DT-109 treatment reduced *NLRP3, IL-1β, and IL-18* expression in macrophages (Supplementary Fig. 8b). It is well known that the NLRP3 inflammasome initiation phase initiates transcription of inflammasome components through the NF-κB signaling pathway. We hypothesize that DT-109 may play a role in inhibiting NLRP3 by influencing NF-κB. Subsequently, the levels of p65 and *p*-p65 were detected by Western blot, and the results showed that the expression of *p*-p65 was decreased after DT-109 treatment (Supplementary Fig. 9a). Therefore, DT-109 may inhibit inflammation by affecting the *p*-p65 signaling pathway.

Corresponding with the coronary transcriptome results, DT-109 suppressed oxidative stress-related genes. Real-time PCR analyses showed that DT-109 downregulated the oxidative stress-related genes *NCF2*, *NCF4*, in cynomolgus monkey coronary artery tissue (Supplementary Fig. 9b). Furthermore, in vascular SMCs treated with 0.5 mM DT-109 compared to non-treated cells, DCFH-DA fluorescence analysis indicated significantly lower levels of superoxide (Fig. 5d). Previously, increased GSH formation was reported as a DT-109 treatment antioxidant effect on macrophages.^25^ Therefore, we analyzed if DT-109 affects GSH in vascular SMCs while exerting its antioxidant effects. Whereas GSH levels decreased in vascular SMCs stimulated with ox-LDL, we observed significantly higher GSH levels in DT-109-treated ox-LDL-stimulated cells (Fig. 5e). In living organisms, the degree of oxidative damage is reflected by the level of malondialdehyde (MDA), the end product of free radical-mediated lipid peroxidation.^31^ Whereas MDA levels increased in vascular SMCs stimulated with ox-LDL, we observed significantly lower MDA levels in DT-109-treated ox-LDL-stimulated A7r5 cells (Fig. 5e). To further verify the inhibitory effect of DT-109 on oxidative stress, we next assayed the levels of GSH and MDA in monkey carotid artery. Similar to the observations in vitro, GSH and MDA levels were significantly up- and downregulated, respectively, in the vasculature of DT-109-treated monkeys (Fig. 5f). Together, these in vivo and in vitro data demonstrated that DT-109 inhibited inflammation and oxidative stress.

### DT-109 alleviates ox-LDL-induced osteogenic differentiation in vascular SMCs

**Fig. 6:**
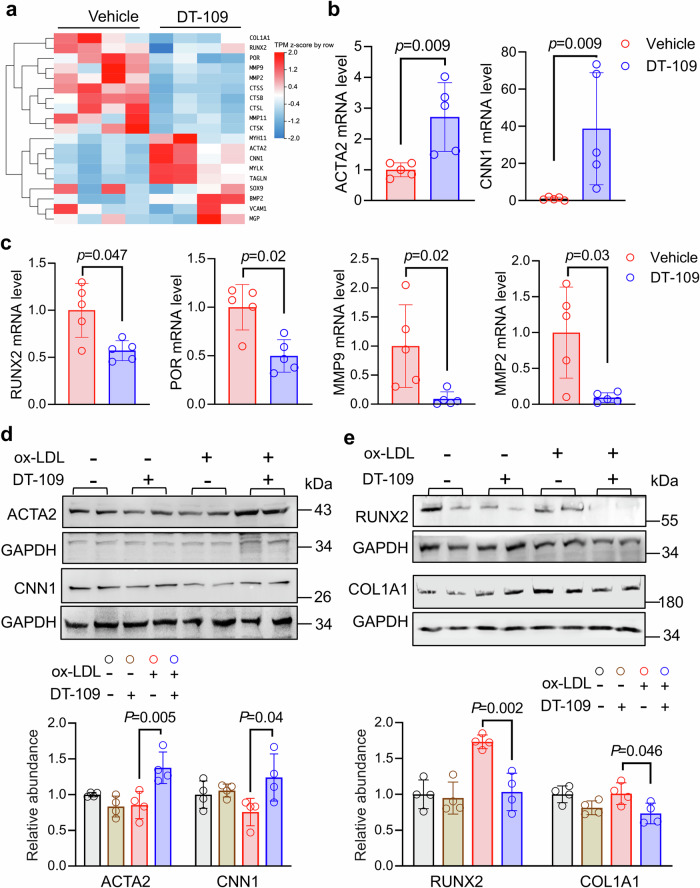
DT-109 alleviates oxidized low-density lipoprotein-induced smooth muscle cell osteogenic differentiation in vitro.**a** Heatmap comparing calcification-related gene expression levels in cynomolgus monkey coronary arteries between vehicle and DT-109 treatment (*n* = 4 for each group). **b** Real-time PCR validation of smooth muscle contraction-related transcription factors actin alpha 2, smooth muscle (*ACTA2*) and calponin 1 (*CNN1*) normalized with 18S in right coronary arteries isolated from vehicle- and DT-109-treated monkeys (*n* = 5 for each group). **c** Real-time PCR validation of vascular calcification-related transcription factors runx family transcription factor 2 (*RUNX2*), cytochrome p450 oxidoreductase (*POR*), matrix metallopeptidase 2 (*MMP2*), and *MMP9* normalized with 18S in monkey coronary arteries (*n* = 5 for each group). **d** Effect of DT-109 on smooth muscle contraction markers (ACTA2 and CNN1) in HASMC and A7r5 cells stimulated with or without ox-LDL for 24 h (*n* = 4 biologically independent samples). **e** Effect of DT-109 on osteogenic differentiation markers (RUNX2 and POR) in A7r5 cells stimulated with or without ox-LDL for 24 h (*n* = 4 for each group). Data are presented as mean ± SD; all data points are shown. Statistical differences were compared using one-way ANOVA with *Tukey’s post hoc* analysis or *Dunn’s* test, multiple comparison adjusted *p*-values were reported

Previously arterial calcification was assumed to be passive and degenerative. Currently, it is considered an active osteogenic process similar to osteoblast differentiation during bone mineralization.^4^ Our histochemistry and micro-CT scan results confirmed that DT-109 reduced calcification and modulated the expression of genes related to osteogenic differentiation in monkey vessels (Fig. 3a–c, Supplementary Fig. 4a–c). Moreover, coronary transcriptome results indicated that DT-109 treatment upregulated vascular smooth muscle contractile pathways (Fig. 4c). Therefore, we analyzed the expression levels of key transcription factors involved in vascular SMCs phenotypic transition and osteogenic differentiation in the monkey coronary transcriptome. Heatmap analysis indicated that DT-109 significantly upregulated smooth muscle contractile markers (e.g., *MYH11*, *ACTA2*, *CNN1*, *MYLK*, and *TAGLN*) and partially downregulated osteogenic transcription factors (e.g., *COL1A1*, *POR*, *MMP2*, *MMP9*, and *MMP11*) (Fig. 6a). Subsequent real-time PCR analysis of right coronary artery tissues confirmed a DT-109-induced increase of the contractile markers (*ACTA2*, *CNN1*, and *TAGLN*) (Fig. 6b, Supplementary Fig. 9c) and a decrease of osteogenic differentiation markers (*RUNX2*, *POR*, *MMP2*, and *MMP9*) (Fig. 6c). However, an apparent inhibition of some osteogenic differentiation-related genes (*SOX9*, *BMP2*, and *MMP11*) was not observed (Supplementary Fig. 9d). To further validate the role of DT-109 in SMC phenotype conversion, we conducted in vitro experiments using ox-LDL-treated HASMC and A7r5 cells. Western blot results showed that DT-109 significantly upregulated smooth muscle contraction markers (ACTA2 and CNN1) (Fig. 6d) and significantly downregulated osteogenic differentiation markers (RUNX2 and COL1A1) (Fig. 6e). Similarly, DT-109 decreased RUNX2 protein levels in ox-LDL-treated HASMCs (Supplementary Fig. 9e). These results suggest that DT-109 inhibits the osteogenic differentiation of vascular SMCs. In summary, our finding suggested that DT-109 inhibits coronary calcification and suppresses SMCs osteogenic phenotype conversion in nonhuman primates.

### DT-109 inhibits SMC osteogenic differentiation

**Fig. 7:**
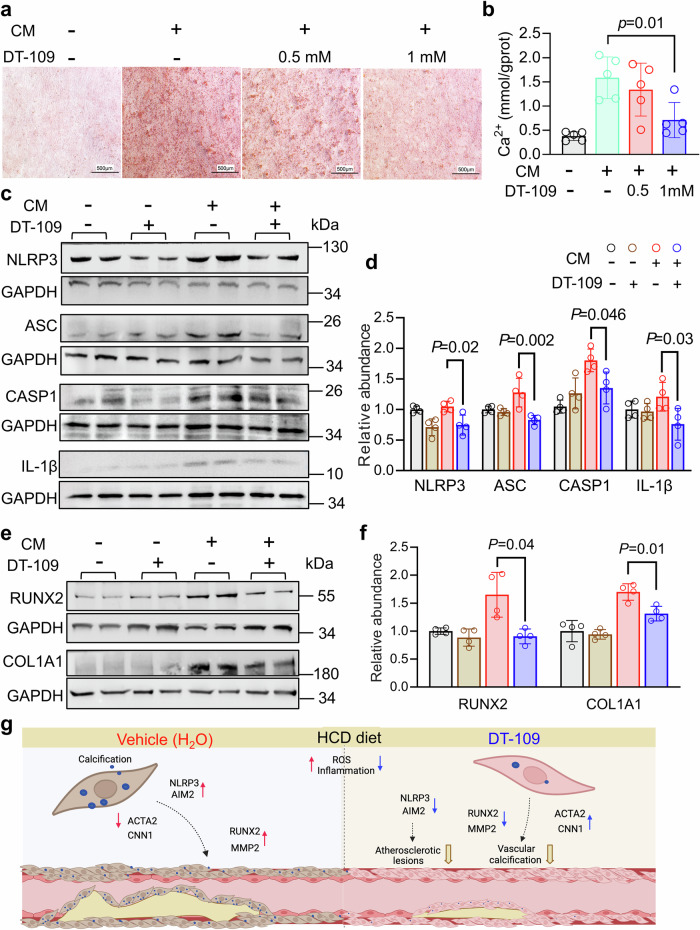
DT-109 inhibits smooth muscle cell osteogenic differentiation.**a** Alizarin red stained images of A7r5 in the vehicle- or DT-109 (0.5 mM and 1 mM)-treated group in response to calcified media (CM) induced for 7 d (Scale bar: 500 μm). **b** Detection of Ca^2+^ concentration in A7r5 cell suspensions after DT-109 (0.5 mM and 1 mM) treatment (*n* = 5). **c** Protein levels of NLR family pyrin domain containing 3 (NLRP3), PYD and CARD domain containing (ASC), apoptosis-related cysteine peptidase (CASP1), and interleukin 1 beta (IL-1β) in A7r5 cells incubated with CM induced for 7 d or basal medium with or without DT-109 (1 mM). **d** Quantitative analysis of protein levels (*n* = 4). **e** Protein levels of runx family transcription factor 2 (RUNX2) and collagen type I alpha 1 chain (COL1A1) in A7r5 cells incubated in CM for 7 d or basal medium with or without DT-109 (1 mM). **f** Quantitative analysis of protein levels *(n* = 4). **g** A schematic illustrating the potential mechanisms by which DT-109 influences atherosclerosis. Online painting: https://app.biorender.com/. Data represent the mean ± SD analyzed by one-way ANOVA with *Tukey’s post hoc* analysis or *Dunn’s* test, multiple comparison adjusted *p*-values were reported

To elucidate the molecular mechanisms by which DT-109 alleviates aortic calcification and inhibits vascular SMC osteogenic phenotype conversion, we performed in vitro SMC culture studies. First, we examined the potential cytotoxicity of calcified medium (CM) on SMC and observed no toxic effects (data not shown). Next, we investigated the impact of DT-109 on SMC migration, contraction, and proliferation under osteogenic differentiation induced by CM. The results demonstrated that DT-109 did not significantly affect SMC migration, proliferation, and contraction in CM conditions (Supplemental Fig. 10a–g). Subsequently, SMCs were treated with DT-109 (0.5 and 1 mM) for 10 days in the presence of normal growth medium or CM, respectively.^32^ We found that DT-109 dose-dependently inhibited vascular SMC calcification assessed by alizarin staining (Fig. 7a), as well as significantly decreased calcium levels in vascular SMCs treated with 1 mM DT-109 (Fig. 7b). Western blot analysis further confirmed decreased protein levels of NLRP3, ASC, CASP1, and IL-1β (Fig. 7c, d) as well as RUNX2 and COL1A1 (Fig. 7e, f), indicating that DT-109 effectively inhibits SMC osteogenic differentiation. However, DT-109 failed to suppress AIM2 levels in calcified vascular SMCs (Supplementary Fig. 11a). Under these conditions, DT-109 no longer affects the abundance of ACTA2 and CNN1 protein and fails to reverse the conversion of an osteogenic to a contractile smooth muscle phenotype (Supplementary Fig. 11b). Although DT-109 reduced the expression of both NLRP3 and RUNX2, the mechanistic link between these two factors remained unclear. To investigate further, we employed a cellular calcification model and found that DT-109 reduced NLRP3 expression even after the cells underwent calcification (Fig. 7c–f), suggesting that NLRP3 may affect the calcification-associated transcription factor RUNX2. Notably, a recent study reported that inhibition or activation of NLRP3 results in reduced or enhanced vascular SMC calcification, respectively.^33^ Therefore, while DT-109 may exert its calcification inhibitory effect partially through the inhibition of NLRP3, but not AIM2, it loses some of its efficacy on the phenotypic transformation of cells that have already acquired osteogenic characteristics.

## Discussion

Recently, DT-109’s therapeutic potential for MASH and cardiovascular-related metabolic disorders has been reported.^25–27^ In the current study, we established a nonhuman primate model of atherosclerosis to evaluate the role of DT-109 in the development of atherosclerosis. Using this unique model, we demonstrated for the first time that oral administration of DT-109 reduces atherosclerotic plaques and macrophage infiltration calcification of lesions in the aorta and coronary arteries. Furthermore, we showed that DT-109 inhibits the inflammatory response and smooth muscle synthetic phenotype switching. Finally, we confirmed the inhibitory effect of DT-109 on the inflammatory response, oxidative stress, and osteogenic differentiation in cultured SMCs (Fig. 7g).

It is challenging to establish calcification and coronary atherosclerosis in mouse experimental model systems.^29^ Cynomolgus monkeys closely resemble the pathophysiology and presentation of disease in humans, therefore they represent a valuable experimental model system.^34,35^ Importantly, cynomolgus monkeys exhibit advanced complicated lesions along with vascular calcification, thus providing a basis for translational research, as shown in this study. Ultimately, a better insight into the molecular mechanism might provide future strategies for the prevention and treatment of calcification while reducing the morbidity and mortality of atherosclerosis-related diseases.^36,37^

It has been reported that plasma glycine is positively associated with the reduced risk of acute myocardial infarction in patients with suspected stable angina.^38^ Moreover, glycine is beneficial in preventing cardiac hypertrophy and heart failure.^39^ While these findings suggest that glycine is involved in reducing the progression of cardiovascular disease, its effect on calcification has not yet been analyzed. In the current study, we first demonstrated that DT-109 inhibited atherosclerotic lesion formation along with calcification in the aorta. Interestingly, blood biochemical analyses show modest reductions in TC, TG, and LDL-C levels within one to two months after DT-109 treatment. However, the AUC for these values did not show a significant difference between the two groups during the 5-month DT-109 treatment. Nevertheless, we can not rule out that this transient change in plasma lipids at early time points may contribute to the inhibition of atherosclerosis and calcification by DT-109 at the study endpoint. Previous studies indicated that DT-109 attenuates MASH through glutathione synthesis and alleviates atherosclerosis through antioxidant effects.^25,26^ Furthermore, reduced concentrations of the reaction substrate GSH facilitate the generation and accumulation of reactive oxygen species (ROS), both hallmarks of calcification.^40^ Based on these studies, we examined the effect of DT-109 on GSH metabolism in monkey arteries and oxidative stress in vascular SMCs and have shown a concentration-dependent DT-109-mediated increase in arterial vascular GSH content as well as a decrease in oxidative stress in vascular SMCs. Clinical outcomes of natural antioxidants are mostly unsatisfactory and not all antioxidants yield favorable benefits. Nevertheless, our findings suggest that DT-109 can act as an antioxidant that exerts a synergistic effect with inhibition of inflammation and suppression of SMC phenotypic switching, ultimately providing protection against atherosclerosis.

It has been well established that atherosclerosis and calcification result from prolonged chronic inflammation.^3,41–43^ To further explore the possible molecular mechanisms of DT-109, we analyzed the coronary transcriptome and demonstrated a significant DT-109-mediated reduction in the inflammatory response signaling pathway. Both NLRP3 and AIM2 are cytoplasmic multiprotein complexes that promote the progression of atherosclerosis. Moreover, inhibition of inflammasome activation was shown to exert effective atheroprotective effects.^44^ Also, activation of NLRP3 was shown to be necessary for vascular SMCs calcification.^45^ Furthermore, glycine-induced inhibition of inflammasome mediators (NALP1, NLRP3, NLRC4, AIM2, and CASP1) was shown to reduce inflammatory cytokine production in macrophages.^46^ However, a glycine-mediated effect on NLRP3 and AIM2 in vascular SMC has not yet been reported. Thus, we demonstrated that DT-109 inhibits ox-LDL-induced activation of inflammation in vascular SMCs in vitro and in vivo. Additionally, we utilized an in vitro model of vascular SMCs calcification to analyze whether DT-109 protects against calcification by preventing inflammation. In this model, we demonstrated DT-109-mediated inhibition of NLRP3 activation restored ACTA2, CNN1, and AIM2 expression levels. Of note, the fact that Canagliflozin, an anti-diabetic medication used to improve blood sugar control in type 2 diabetes, was shown to protect against calcification through inhibition of the NLRP3 signaling pathway further suggests the potential therapeutic value of NLRP3 inflammasome inhibition.^33^

It is important to acknowledge several limitations of the current study that should be addressed in future research for clinical translation. First, the study only included male animals, so it remains unclear whether the beneficial effects of DT-109 extend to females. Second, while the liver plays a critical role in systemic metabolism, it remains unclear whether the observed inhibition of atherosclerosis and calcification with DT-109 treatment is related to any effects on improved liver function. Third, we did not conduct a dose-dependence test on the effects of DT-109 in inhibiting atherogenesis due to resource constraints. Performing such a study would help identify the optimal dosing strategy for maximizing its therapeutic benefits. Additionally, although the monkeys were housed in individual cages with access to food and water *ad libitum*, we were unable to measure food intake due to the lack of metabolic cages. Furthermore, blood pressure was not monitored during the experiment, which is another aspect to consider in future studies. Finally, the monkeys were fed an HCD to induce hypercholesterolemia and atherosclerosis, as it is challenging to develop atherosclerosis spontaneously in these animals due to their shorter lifespan and lower plasma LDL-C levels compared to humans. Future studies should also clarify whether the beneficial effects of DT-109 are observed in normocholesterolemic conditions.

In conclusion, our findings demonstrated that the glycine-based tripeptide DT-109 not only reduces aortic and coronary atherosclerotic lesions but also ameliorates calcification in a nonhuman primate model. These beneficial effects are apparently attributed to DT-109’s ability to attenuate inflammation and oxidative stress. Specifically, our findings suggest that DT-109 is capable of inhibiting the activation of inflammatory pathways and reducing oxidative damage within the vascular system. As such, these results provide a new potential therapeutic avenue for the treatment and prevention of atherosclerosis, a disease characterized by chronic inflammation and oxidative injury to the arterial walls.

## Materials and Methods

### Experimental Design

Twenty male cynomolgus monkeys were fed an HCD for 10 months to induce atherosclerotic lesions. Using a stratified randomization process based on their NAS scores, the monkeys were randomly divided into two groups.^27^ They were given DT-109 (150 mg/kg, dissolved in H_2_O, Diapin Therapeutics) or vehicle (same volume of H_2_O) by gavage daily for 5 months with continuation of the HCD.^27^ The monkeys were anesthetized and euthanized as the previous report.^27^ The aorta and major branches were fixed in 10% buffered formalin while a portion of the common carotid artery was stored in liquid nitrogen for RNA extraction or ultrasonically fragmented for supernatant extraction (Supplementary Fig. 12a). The coronary arteries were isolated, four right coronary arteries in each group were used for RNA-seq analysis, and the remaining right coronary arteries were snap-frozen with liquid nitrogen, then extract RNA or protein for further data validation. Left coronary arteries were fixed with 10% buffered formalin for pathologic examination and comparison.

### Cynomolgus monkey studies

All animal studies were approved by the Laboratory Animal Care Committee of Xi’an Jiaotong University (No. 2019-1278) and approved by the Institutional Animal Care and Use Committee of Spring Biotechnology Development Company Limited (No. 201901). Housed in a cycling facility at 22 ~ 26 °C with a 12 h light cycle / 12 h dark cycle. The study was performed in accordance with the Guide for the Care and Use of Laboratory Animals by the National Institutes of Health (8th edition, 2011).^27^

### Analysis of atherosclerotic lesions

The entire aorta trees were opened and stained with Sudan IV (Solarbio, China) to measure the gross lesion area. In brief, the aorta was removed from the formalin and washed with running water for 1 h. Sudan IV staining was performed after 30 minutes of differentiation in 70% alcohol, followed by washing with running water for 1 hour^47^ The area of the atherosclerotic lesion (Sudanophilic area) was measured using image analysis software to calculate the proportion of plaque to the area of the entire aorta (WinROOF 6.5, Mitani, Tokyo).

To quantify the microscopic lesions, the aortic arch was cut into 5 μm thick sections.^48^ The coronary arteries were separated from the heart and stored in 10% buffered formalin solution. As shown in Supplementary Fig. 12b, the left circumflex (LCX) artery was cut into four segments (approx. 500 µm in length, labeled 1–4 as in the figure). 10 serial sections of each segment were sectioned (5 µm in thickness) and used for pathological analysis and comparisons between the two groups.

For histological analysis, the sections were stained with H&E (Thermo Fisher Scientific), Elastica van Gieson (EVG, Solarbio, China) and Masson’s trichrome staining (Solarbio, China). For immunohistochemistry analysis, paraffin sections were heated for 10 min in ethylene-diamine-tetra-acetic acid buffer (Beyotime, China) for antigen retrieval and then placed in 3% hydrogen peroxide for 15 min. Sections were then pre-incubated with 2% horse serum (Proandy, China) for 2 h followed by incubation with primary antibodies against human macrophages (CD68) (Sigma-Aldrich, USA) and smooth muscle actin-alpha 2, (ACTA2, Sigma, USA) overnight at 4 °C. Next, samples were washed and incubated with a secondary antibody against rabbit IgG (Proteintech, USA) for 1 h at room temperature. Target proteins were visualized using a DAB substrate kit (Zsgb-bio, China) and counterstained with hematoxylin. Histopathological images were captured under a light microscope (Olympus, Japan) and quantified using WinROOF 6.5.

### Analysis of vascular calcification

Von Kossa staining (Solarbio, China) was used to determine the mineral nodules of vascular tissues. Briefly, slides were dehydrated and incubated with 1% silver nitrate solution under ultraviolet light for 30 min, and then the slides were placed in 5% sodium thiosulfate distilled water for 10 min, and the calcified nodules were stained black.

Micro-CT (Quantum GX2, PerkinElmer) scan of the thoracic aortic portion was performed to detect the presence of calcified lesions in the aorta followed by a statistical analysis of the area of calcification.

### Analysis of calcium content

For quantification, the supernatant was centrifuged at 2500 g for 10 min after carotid artery tissues homogenization. Then, the MTB reagent, alkaline solution, and protein clarifier were added according to the manufacturer’s instructions (Njjcbio, China). Finally, the absorbance of the samples was read at 610 nm with a plate reader and normalized to the protein content determined using a bicinchoninic acid protein assay (ZHHC, China).^49,50^

### Analysis of glutathione and malondialdehyde

Carotid artery tissues were rapidly removed from the euthanized monkeys, frozen in liquid nitrogen, and kept at -80 °C. The amount of GSH was determined using an enzyme assay kit (Njjcbio, China) according to the manufacturer’s protocol. In brief, after weighing the tissue samples, nine times the volume normal saline was added for ultrasonic crushing. The samples were centrifuged at 2500 g for 10 min after which the supernatant was taken for measurement. Then, the cells were collected, supplemented with 0.5 mL PBS, and lysed by sonication. The same volume of reagent was added and mixed according to the instructions. After the centrifugation, the supernatant was taken to quantitatively determine reduced GSH.

The amount of MDA was determined using an enzyme assay kit (Njjcbio, China). The supernatant of cells and tissues lysed during GSH detection was used and processed according to the manufacturer’s recommended protocol.

### Quantitative real-time PCR

Total RNA was extracted from monkey coronary arteries using Trizol (Accurate Tech, China) and equal amounts were reverse transcribed into cDNA. In brief, an appropriate amount of Trizol according to the tissue size was added for homogenization and then centrifuged at 12,000 g for 10 min at 4 °C. The supernatant was transferred to a new tube with 0.2 ml chloroform and centrifuged at 12,000 g for 10 min at 4 °C. The supernatant was collected, 0.2 ml of isopropyl alcohol was added centrifuged, and the precipitate was washed with 1 ml 70% ethanol. An appropriate amount of distilled H_2_O was added to dilute RNA for quality control analysis. The SYBR Green 2X PCR mix (Accurate Tech, China) was used for quantitative real-time PCR amplification. All results was normalized to 18S. The specificity of the resulting PCR products was confirmed by melting curve analysis. Relative gene expression was measured by the comparative Ct method, X = 2^−ΔΔCt^. Primer sequences are listed in the Supplementary Table 1.

### RNA-sequencing (RNA-seq) analysis of coronary arteries

At the end of the experiment, the right coronary arteries were isolated from the heart for RNA sequencing analysis. The RNA libraries were constructed according to the instructions provided by the Beijing Genomics Institute (BGI, Shenzhen, China).^51,52^ The qualified libraries were sequenced on the DNBSEQ system at BGI. Briefly, total RNA samples were subjected to DNAzyme I treatment to synthesize cDNA strands, and the amplified cDNA ends were repaired. PCR reaction is performed on the ligation product and the PCR product is subsequently cyclized to obtain a single-stranded circular DNA library. Single-stranded circular DNA molecules form DNA nanospheres for on-machine sequencing. After quality control of the RAW reads, clean reads obtained by filtering were compared with the reference sequence.

Publicly available gene expression datasets from patients with early and advanced atherosclerotic plaque samples were used to compare transcriptome profiles between the nonhuman primates and humans. The study included RNA array data from 13 early plaque samples and 16 advanced plaque samples from patients with atherosclerosis (GSE28829).^30^ The similarities and differences between cynomolgus monkey and human transcriptome data in the Kyoto Encyclopedia of Genes and Genomes (KEGG) pathway were analyzed using GSEA analysis software.

### THP-1 monocyte culture and induced differentiation

THP-1 cell lines were maintained in RPMI-1640 (HyClone, USA), 10% FBS, and 100 units/mL penicillin and 100 μg/mL streptomycin (HyClone, USA) at 37 °C under a humidified atmosphere of 5% CO_2_. THP-1 cells were induced with PMA (Sigma, USA) 100 ng/ml for 48 h to induce monocyte differentiation into macrophages.

### Vascular SMC culture and calcification induction study

A7r5 rat thoracic aortic SMCs were maintained in DMEM supplemented with high glucose (HyClone, USA), 10% FBS, and 100 units/mL penicillin and 100 μg/mL streptomycin (HyClone, USA) at 37 °C under a humidified atmosphere of 5% CO_2_. HASMCs were cultured in DMEM/F-12 (Sigma, USA) supplemented with 15% FBS, 100 units/mL penicillin, and 100 μg/mL streptomycin at 37 °C in a humidified atmosphere with 5% CO_2_. HASMCs were used within ten generations. Cells were stimulated with ox-LDL (50 μg) with or without DT-109 for 24 hours.

A7r5 cells were incubated with calcifying medium [DMEM containing high glucose (HyClone, USA), 15% FBS (ThermoFisher, China), 100 units/mL penicillin and 100 μg/mL streptomycin (ThermoFisher, China), 15 mM sodium beta-glycerophosphate (Sigma, USA), and 0.35 mM L-ascorbic acid (Sigma, USA)]. Cells were cultured in the calcifying medium for 5 or 10 days with medium changes every 3 days to induce calcification. After reaching confluency (70–80%), cells were cultured with calcifying medium with or without DT-109 for 5 days for Western blot analysis or 10 days for subsequent staining and calcium content determination.

A7r5 cells were cultured in the calcifying medium for 10 days and then washed three times with PBS after which calcium deposition was visualized and quantified by Alizarin Red (Solarbio, China) staining as recommended by the manufacturer. In brief, cells were fixed in 4% formaldehyde for 15 min at room temperature and washed three times with distilled H_2_O. Moderate Alizarin Red was added per well and incubated at room temperature for 30 min. After washing the cells five times with distilled H_2_O, images were taken with a camera.

### Vascular SMC migration, contraction, and proliferation assays

#### Cell migration by wound-healing assay

MOVAS cell lines (AC338213; ATCC) were seeded in 6-well plates. The cells were then wounded by manual scraping with a 100 μL micropipette tip. After washing the cells once with the desired medium to remove any floating cells, they were treated with ox-LDL (50 μg/ml) with or without DT-109 (0.5 mM and 1 mM) for 24 h in a serum-free medium. Images were captured at 0 h and 24 h using a microscope at the same region. Quantitative analysis was performed using ImageJ.

#### Cell contraction by collagen gel contraction assay

HASMC cells (AC339826; ATCC) were mixed with type I collagen solution (1.2 mg/mL, Cell Biolabs Inc., cat#CBA-201) according to the manufacturer’s protocol. The mixture was added to 24-well tissue culture plates (2.5 × 10^6^ cells in 0.5 ml/well) and allowed to form a gel at 37 °C for 1 h. Then, 1 mL of culture medium was added to each well. After 24 h, the edges of the collagen gel were mechanically lifted, and images were taken using a microscope at various time points. The cells were pretreated with DT-109 for 12 h, followed by ox-LDL/TGF-β (10 ng/mL) stimulation for 24 h. The reduction in gel surface area was measured using ImageJ to assess cell contraction. Imaging and data analyses were performed blindly.

#### Cell proliferation assessed by Ki-67 staining

HASMC cells (AC339826; ATCC) were cultured in the SMC growth medium (Lonza, CC-3182) containing 5% fetal bovine serum (Lonza, Switzerland) and 1% penicillin/streptomycin solution at cell culture incubator. When the cells reached 70% confluence, they were treated with either vehicle or DT-109 (1 mM) for 12 h, followed by ox-LDL (50 μg/ml) for 24 h. Please refer to the previous article for details.^53^

### Reactive oxygen species assay

The quantity of ROS was measured with 2,7-dichlorodihydrofluorescein diacetate (DCFH-DA). Cells (70–80% confluency) were incubated 24 h in advance with different working concentrations of DT-109. Then, ox-LDL (50 μg/ml in serum-free medium) was added, and cells were further incubated at 37 °C for 2 h in a cell incubator protected from light. Next, DCFH-DA (10 μM in serum-free medium) was added, and the cells were further incubated for 30 min at 37 °C in a cell culture incubator protected from light. Finally, cells were washed with serum-free culture medium and the fluorescence signal was detected by fluorescence microscopy at a maximum excitation wavelength of 480 nm and a maximum emission wavelength of 525 nm.

### Western blot analysis

Cellular proteins were extracted with RIPA lysis buffer (ZHHC, China) and quantified by BCA. Proteins (30 μg/lane) were separated by sodium dodecyl sulfate-polyacrylamide gel electrophoresis and transferred to PVDF membranes. After being incubated with 5% milk for 2 h at room temperature, they were incubated with primary antibodies overnight at 4 °C. After incubation with horseradish peroxidase-conjugated secondary antibody (Proteintech, USA) for 2 h at room temperature, the signals were visualized using an ECL kit (Millipore, USA). Relative protein abundance was normalized to GAPDH and quantified using Image J.

### Statistical analyses

We conducted the *Shapiro–Wilk* test to assess the normality of each dataset and *Levene’s* test to examine the equality of variances across multiple groups. For comparisons between two groups, the *Kruskal–Wallis* test was utilized. When the assumptions of normality and equal variance were satisfied, *Tukey’s Honestly Significant Difference (HSD)* test was applied for pairwise comparisons across multiple groups. In cases where variances were unequal, *Dunn’s* test was used for nonparametric post-hoc groupwise comparisons. Adjusted *p-*values from *Tukey’s HSD* and *Dunn’s* tests were reported to account for multiple tests. Data are represented as the mean ± standard deviation (SD). Statistical significance was set at a *p*-value of 0.05. All statistical analyses were performed using R (The R Project for Statistical Computing), version 4.4.1.

## Supplementary information

SIGTRANS-12160R2_Suppl info

## Data Availability

All data supporting the findings of this research can be obtained from the corresponding authors upon a reasonable request. RNA-sequencing data generated from coronary arteries in this study were deposited to the National Center for Biotechnology Information (NCBI) Sequence Read Archive (SRA) database with accession number PRJNA1016068.The antibodies and reagents used in the current study can be found in Supplementary Table 2. The list of abbreviations is in Supplementary Table 3.
