# Supplementary material for: Tripeptide DT-109 (Gly-Gly-Leu) attenuates atherosclerosis and vascular calcification in nonhuman primates
Source: Signal Transduct Target Ther. 2025 Apr 7;10:122. doi: 10.1038/s41392-025-02201-2 (PMC11977015; doi:10.1038/s41392-025-02201-2)
Supplement: Supplementary file 1 — SIGTRANS-12160R2_Suppl info [file 41392_2025_2201_MOESM1_ESM.docx]

**Supplementary Materials for**

**Tripeptide DT-109 (Gly-Gly-Leu) attenuates atherosclerosis and vascular calcification in nonhuman primates**

Linying Jia^1,#^, Pengxiang Qu^1,#^, Yang Zhao^2^, Liang Bai^1^, Honghao Ren^1^, Ao Cheng^1^, Zeyao Ma^1^, Cheng Ding^1^, Yongjie Deng^2^, Lingxuan Kong^3^, Ying Zhao^2^, Oren Rom^2, 4^, Yajie Chen^5^,  Naqash Alam^1^, Wenbin Cao^1^, Sixue Zhai^6^, Zuowen Zheng^7^, Zhi Hu^8^, Lu Wang^3^, Yabing Chen^9^, Sihai Zhao^1^, Jifeng Zhang^2,*^, Jianglin Fan^5,*^, Y. Eugene Chen^2,*^, Enqi Liu^1, 10, 11,*^

*Corresponding authors. Email: [liuenqi@mail.xjtu.edu.cn](mailto:liuenqi@mail.xjtu.edu.cn) (Enqi Liu); [echenum@umich.edu](mailto:echenum@umich.edu) (Y. Eugene Chen); [jianglin@yamanashi.ac.jp](mailto:jianglin@yamanashi.ac.jp) (Jianglin Fan).

^#^ Linying Jia and Pengxiang Qu contributed equally.

This file includes:

Supplementary Figures. 1 to 11 (pages 2-20)

Supplementary Tables 1 to 3 (pages 21-24)

Uncropped Western blot images (pages 25-33)

**
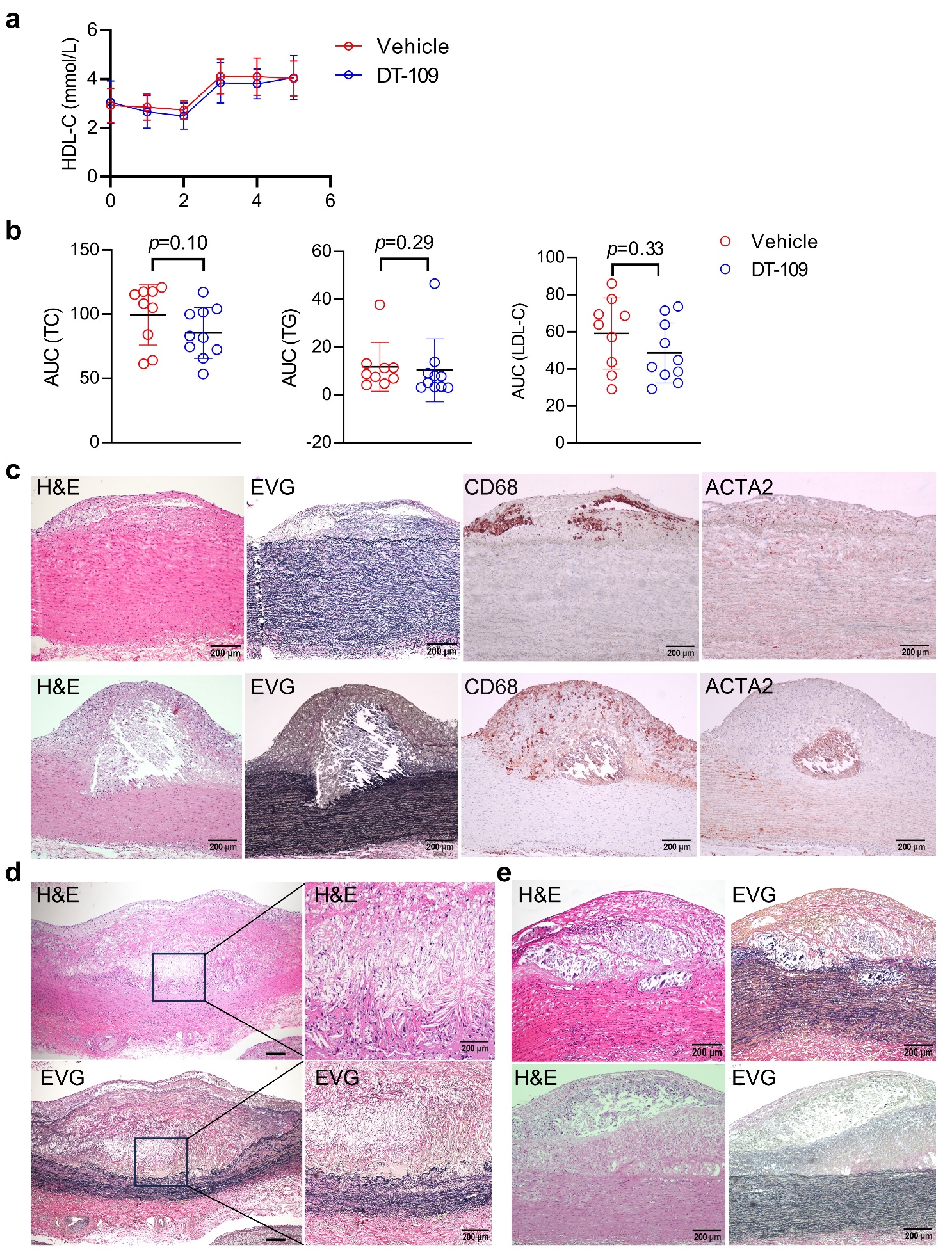
**

**Supplementary Fig. 1 Plasma lipid levels and plaque characterization.**

**(a)** Plasma high-density lipoprotein cholesterol (HDL-C) levels in cynomolgus monkeys (male, age ≥ 9 years, BMI ≥ 30) fed an HCD for 10 months and randomized to receive either DT-109 (150 mg/kg/d) or vehicle for an additional 5 months (n=10 for each group). **(b)** Area under the curve (AUC) of changes in TC, TG, and LDL-C levels in monkeys after 5 months of DT-109 treatment. Data are presented as mean ± SD at each time point. All statistical comparisons between groups were evaluated by *Kruskal–Wallis* test. Vehicle, n=9; DT-109, n=10. (c) Intermediate and advanced plaque lesions of the aortic arch. **(d)** Typical pathologic features of cholesterol crystals (arrow symbol). Atheromatous plaques are typically characterized by a superficial fibrous cap, and deeper by an atheromatous substance consisting of a mixture of lipids and necrotic disintegrating material, which is rich in cholesterol crystals. The smooth muscle of the middle membrane was atrophic and thin. Neoplastic capillaries, connective tissue hyperplasia, and lymphocyte and plasma cell infiltration were seen in the outer membrane. **(e)** In a combined lesion based on fibrous plaque and atheromatous plaque, also known as a composite lesion. Calcium salts are deposited in the atheromatous necrotic material and within the fibrous cap, causing the arterial wall to become hard, brittle, and may ossify.

**
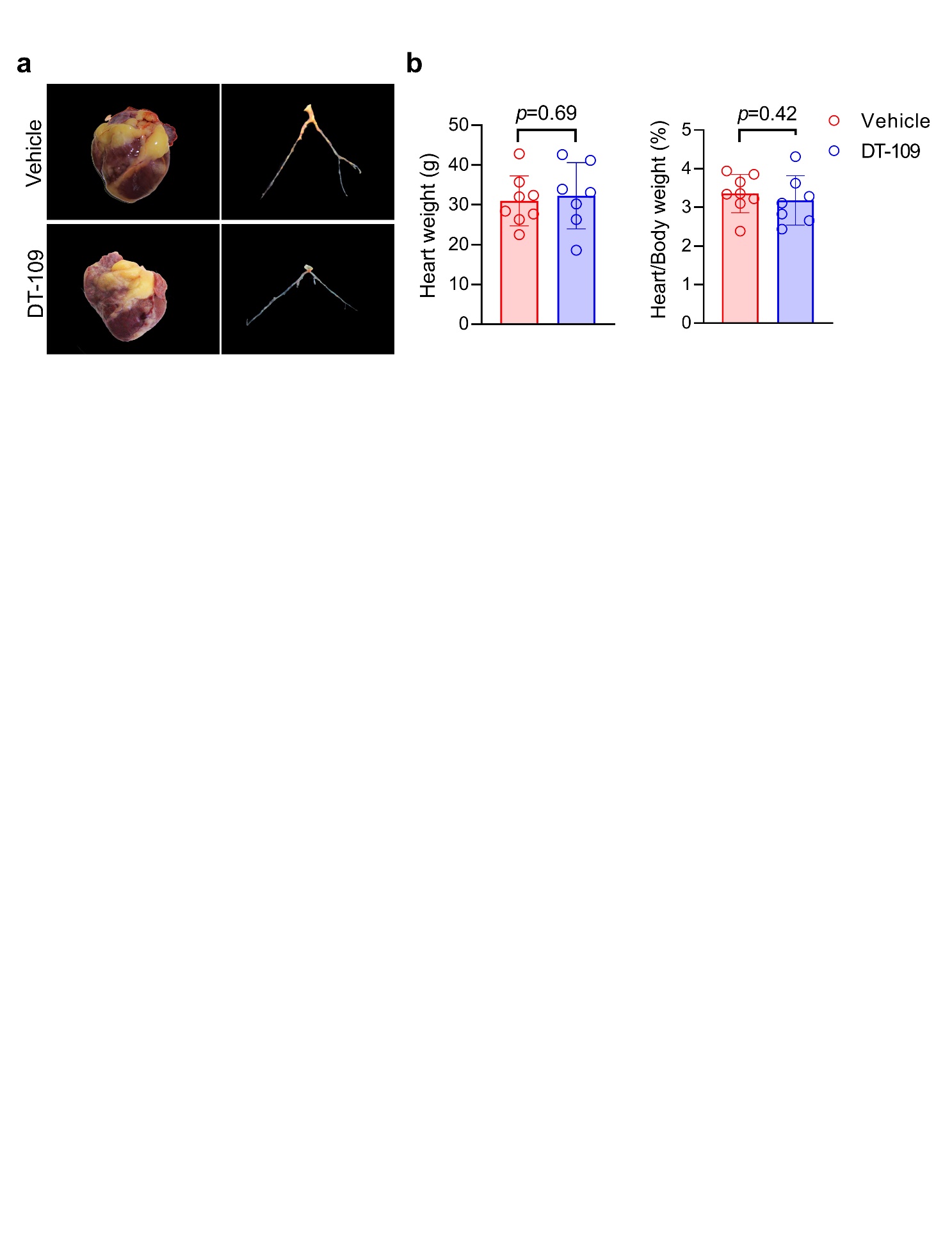
**

**Supplementary Fig. 2 Monkey body weight and heart weight.**

**(a)** Monkey heart and isolated left coronary artery in a holistic view. **(b)** Quantification of heart weight and heart-to-body weight ratio in cynomolgus monkeys (male, age ≥ 9 years, BMI ≥ 30) fed an HCD for 10 months and randomized to receive either DT-109 (150 mg/kg/d) or vehicle for 5 an additional 5 months (vehicle, n=8; DT-109, n=7). Data are presented as mean ± SD. All statistical comparisons between groups were evaluated by *Kruskal–Wallis* test.

**
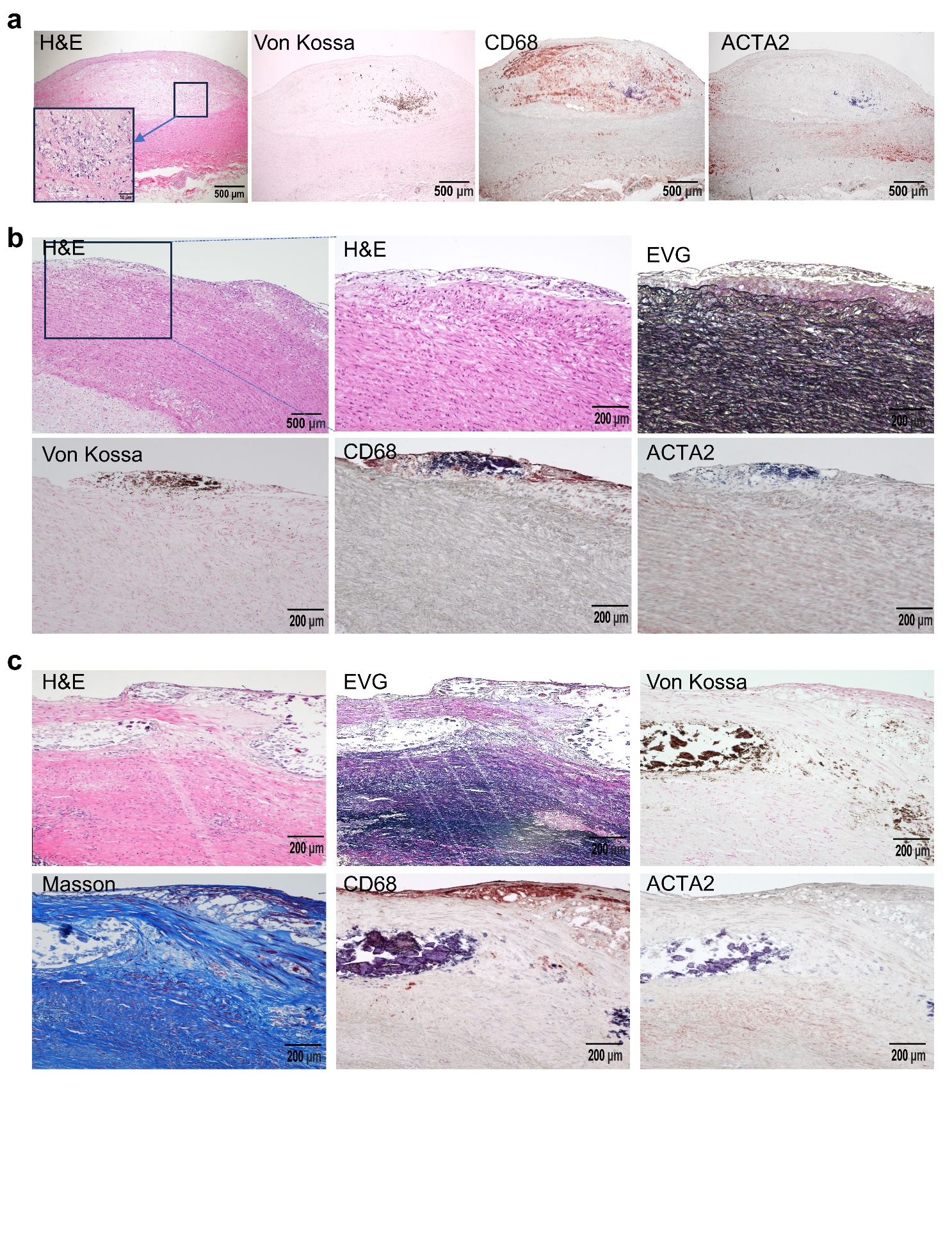
**

**Supplementary Fig. 3 Analysis of vascular calcification.**

**(a)** Typical lesion features of micro-calcification visualized by Von Kossa staining. **(b)** Endothelial calcified lesions.Typical features of unstable plaque calcification. **(c)** Typical lesions of mesangial calcification.

**
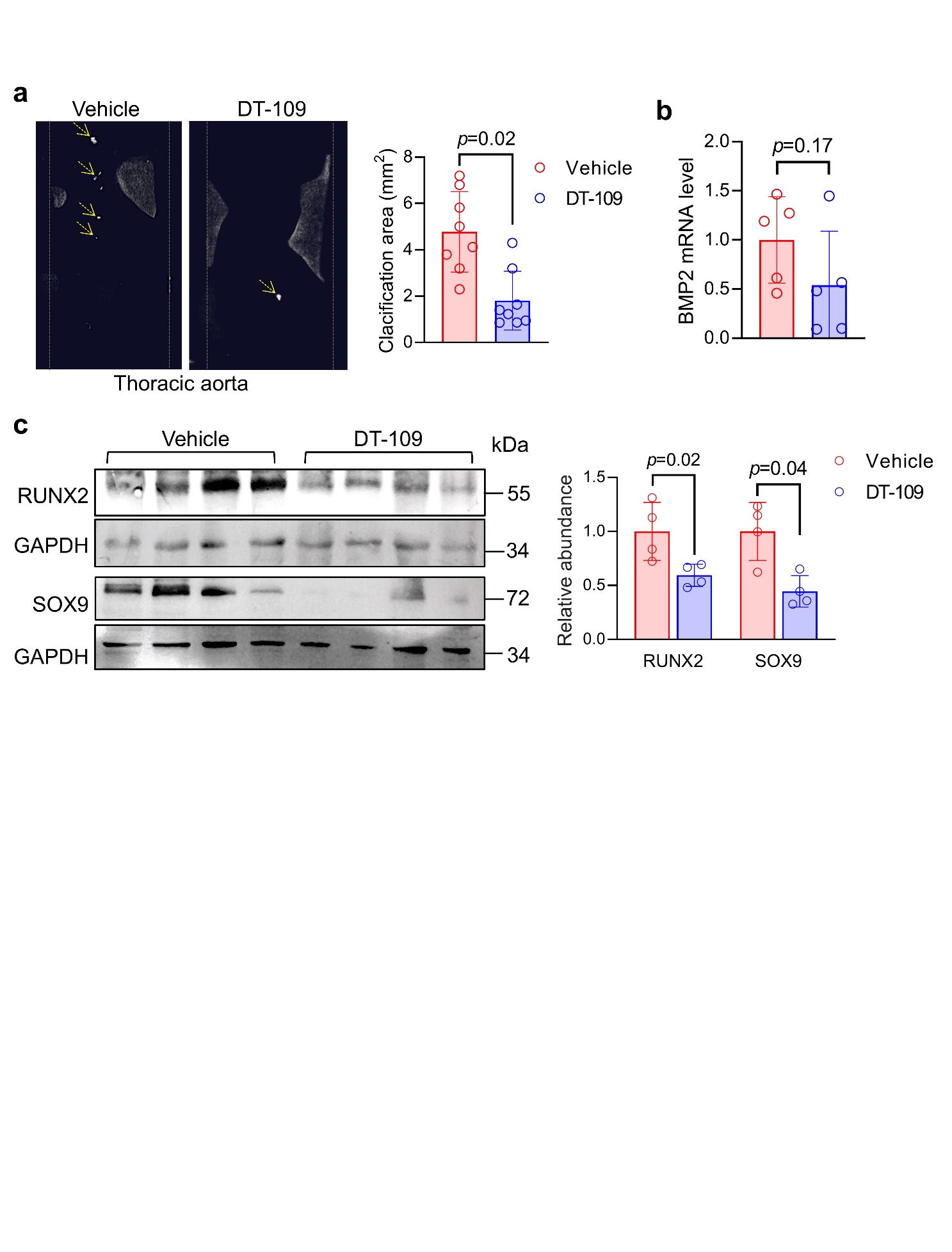
**

**Supplementary Fig. 4 Effect of DT-109 on vascular calcification *in vivo*.**

**(a)** Thoracic aorta micro-CT scan, statistical analysis of the calcified spots area (vehicle, n=8; DT-109, n=8). **(b)** Extraction of total RNA from monkey common carotid arterial tissue and detection of bone morphogenetic protein 2 (BMP2) mRNA expression by real-time PCR (n=5 for each group). (**c**) Detection of RUNX family transcription factor 2 (RUNX2) and SRY-Box transcription factor 9 (SOX9) protein levels in monkey common carotid artery tissues (n=4 for each group). Quantification of protein levels. Data are presented as mean ± SD. All statistical comparisons between groups were evaluated by Kruskal–Wallis test.

**
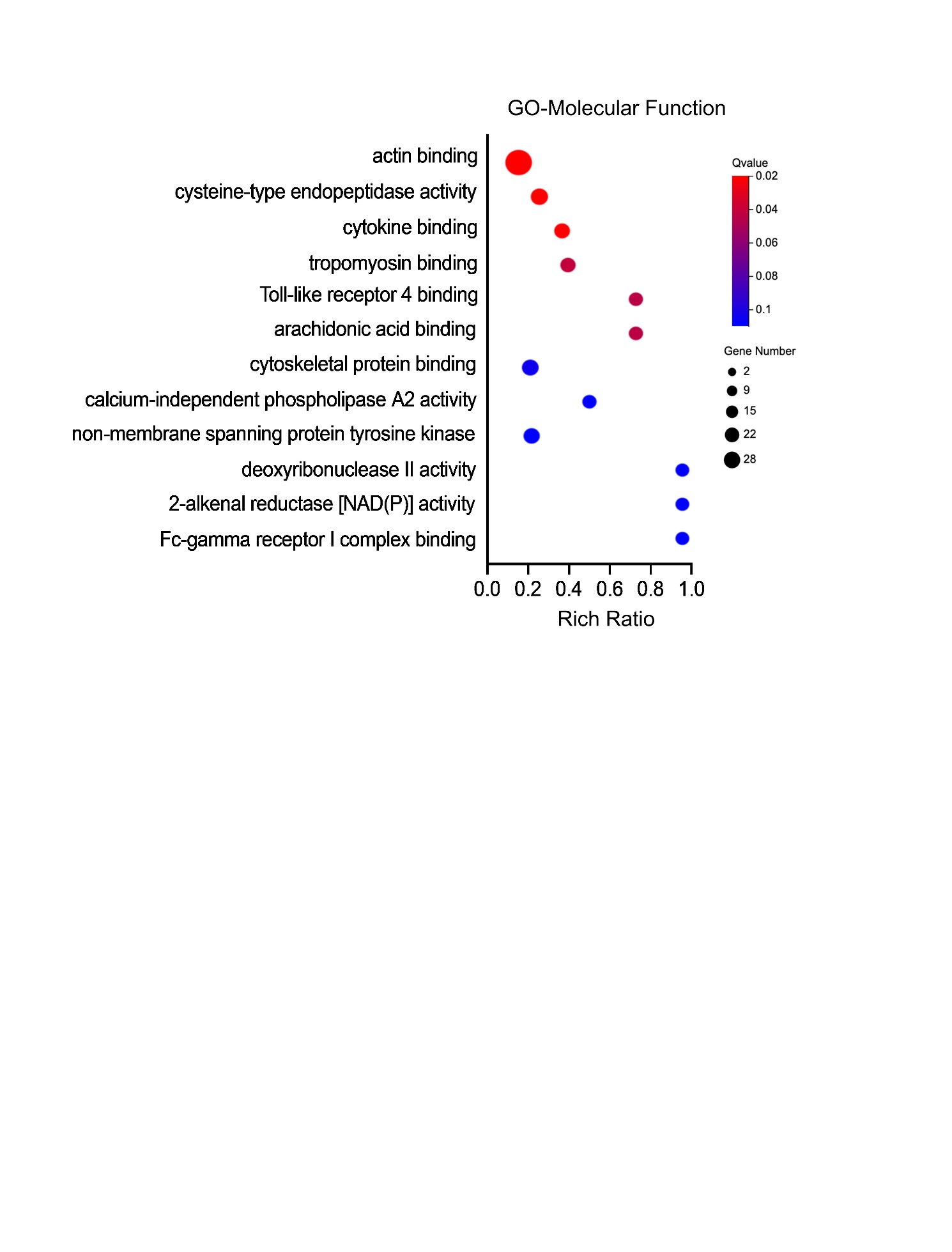
Supplementary Fig. 5 Coronary transcriptome GO (gene ontology) analysis_GO-F (molecular function).**

**
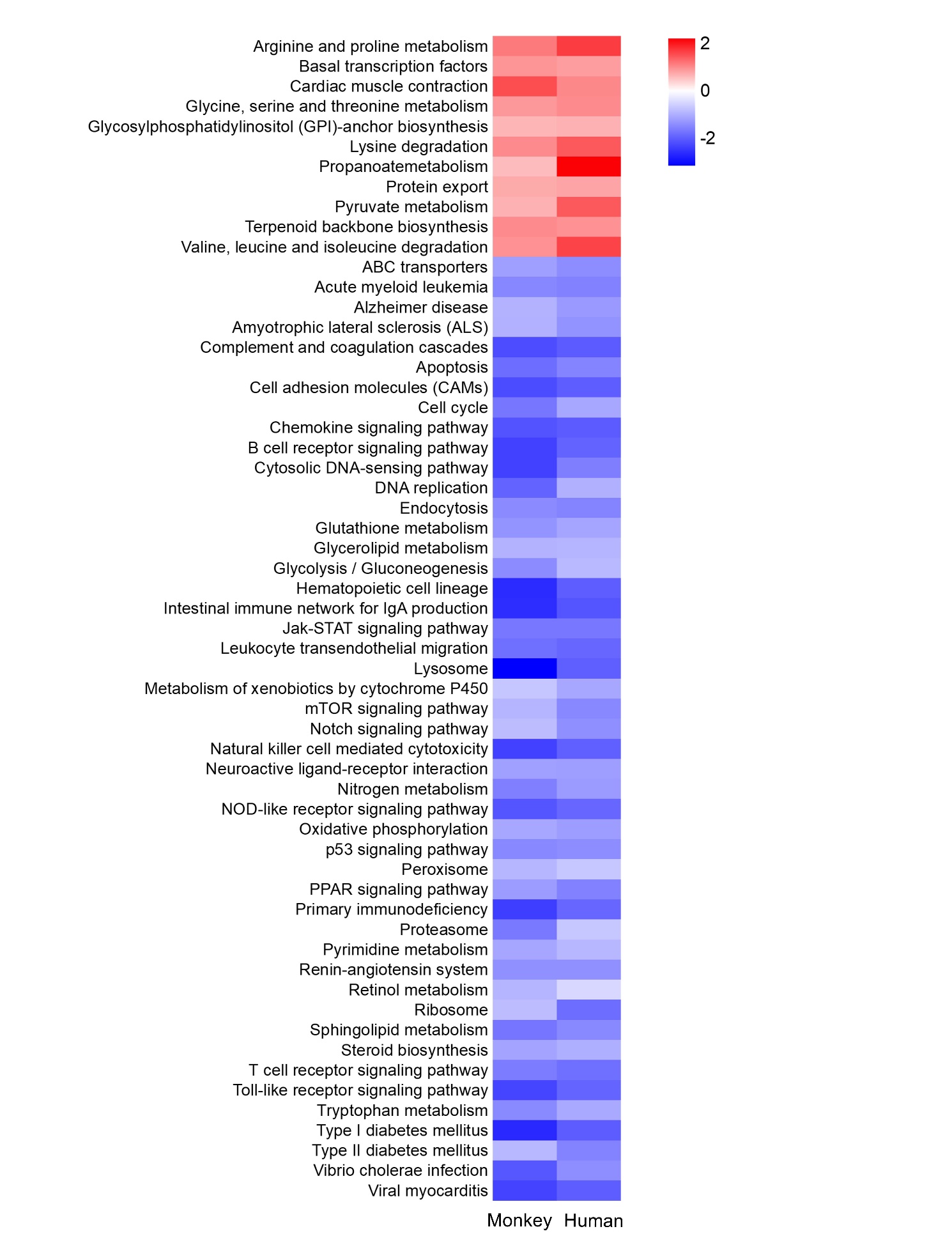
**

**Supplementary Fig. 6 Comparative analysis of differential gene pathway enrichment in monkey and human atherosclerotic plaques.**

Comparison between post-atherosclerotic cynomolgus monkeys treated with DT-109 and human early and advanced atherosclerotic plaques to identify similarities and differences between Kyoto Encyclopedia of Genes and Genomes (KEGG) pathways. The study included RNA-seq data from 13 early plaque samples and 16 advanced plaque samples from patients with atherosclerosis (GSE28829).

**
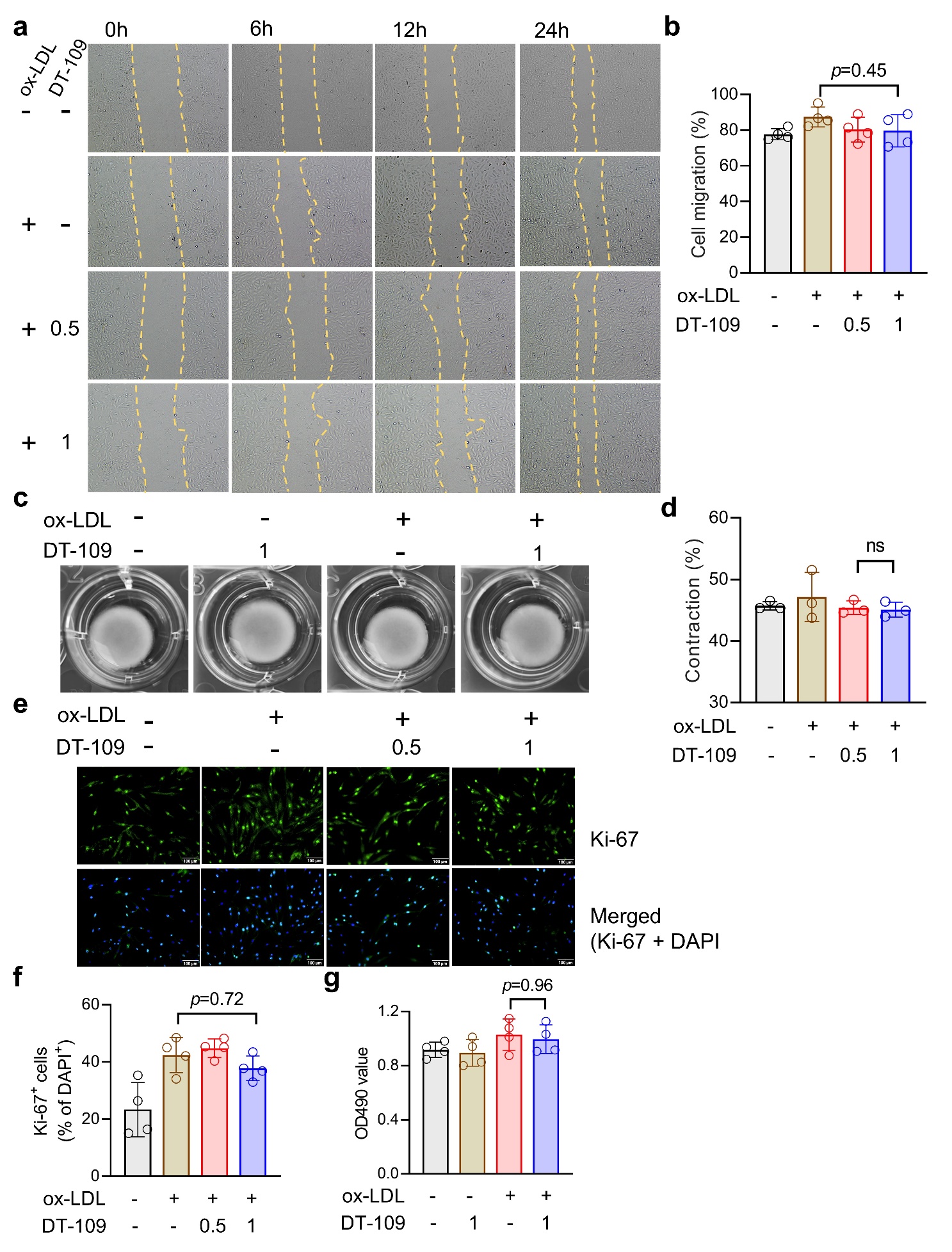
**

**Supplementary Fig. 7 DT-109 has no significant effect on SMC migration, contraction, and proliferation in response to ox-LDL stimulation.**

**(a-b)** The DT-109 administration group was pretreated for 12 h, and ox-LDL (50 μg/ml) was stimulated for 24 h. Scratch assay was performed to detect the migration of the cells within 24 hours (n=4). **(c-d)** The cells were pretreated with DT-109 for 12 h and then stimulated with ox-LDL (50 μg/ml) for 24 h. A collagen gel contraction assay was used to evaluate cell contraction (n=4). **(e-f)** The administered group DT-109 was pretreated for 12 h before ox-LDL (50 μg/ml) stimulation for 24 h. Then Ki-67 fluorescence staining was performed and quantified (n=4). **(g)** The administered group DT-109 was pretreated for 12 h before ox-LDL (50 μg/ml) stimulation for 24 h, and then the absorbance was measured after CCK-8 treatment for 30 min (n=4). Data are represented as mean ± SD. Statistical differences among multiple groups were compared using one-way ANOVA with *Tukey’s post hoc* analysis or *Dunn’s* test, multiple comparison adjusted *p*-values were reported.

**
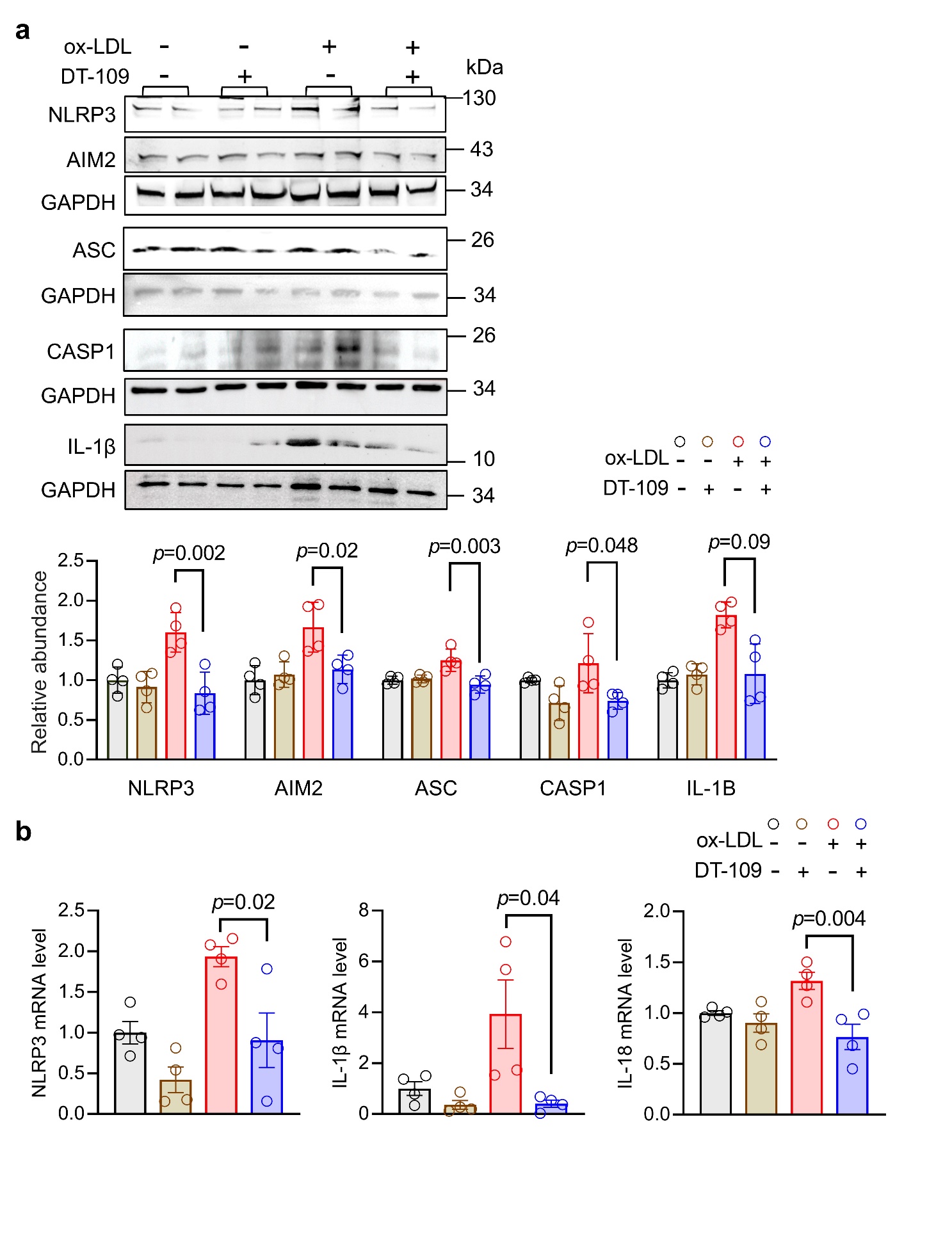
**

**Supplementary Fig. 8 DT-109 inhibits ox-LDL-induced vascular smooth muscle cell inflammation.**

**(a)** Human aortic smooth muscle cells (HASMCs) were treated with ox-LDL for 24 h, then protein was extracted. Western blot was performed to detect the protein levels of NLR family pyrin domain containing 3 (NLRP3), absent in melanoma 2 (AIM2), PYD and CARD domain containing (ASC), apoptosis-related cysteine peptidase (CASP1), and interleukin 1 beta (IL-1β) in DT-109-treated human aortic smooth muscle cells (n=4). (**b**) Expression levels of *NLRP3, IL-1β, IL-18* after DT-109 treatment in THP-1 cell line (n=4). Data are represented as mean ± SD. Statistical differences among multiple groups were compared using one-way ANOVA with *Tukey’s post hoc* analysis or *Dunn’s* test, multiple comparison adjusted *p*-values were reported.

**
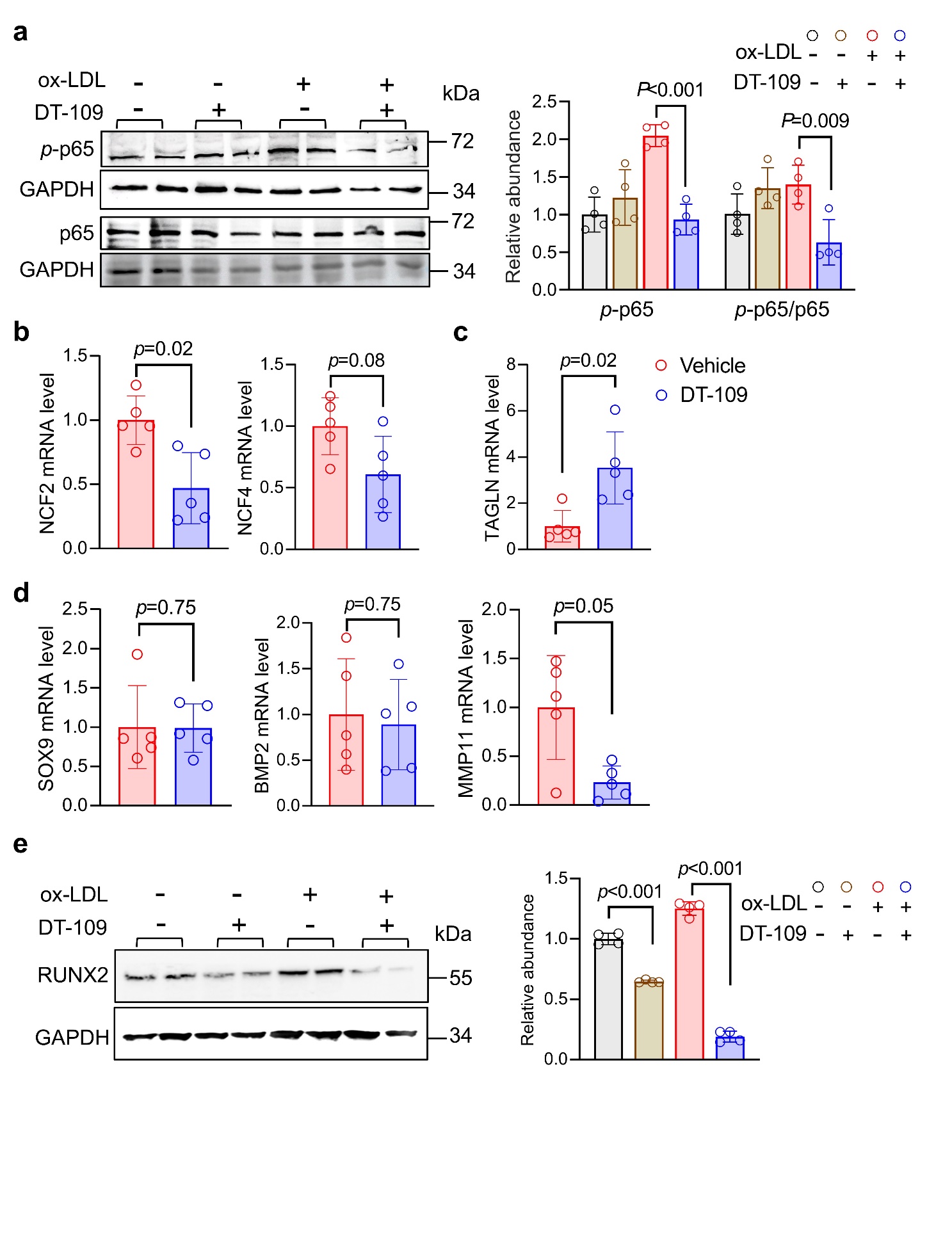
Supplementary Fig. 9 Effects of DT-109 on smooth muscle cell inflammation, oxidative stress, and osteogenic differentiation.**

**(a)** DT-109 inhibits levels of NF-kB p65 phosphorylation. **(b)** Gene expression of *neutrophil cytosolic factor 2* (*NCF2*) and *NCF4*in monkey common carotid arteries tissue (n=5). **(c)** Real-time PCR detected DT-109-induced elevated expression levels of the contraction marker *transgelin* (*TAGLN*) in right coronary artery tissue (n=5). **(d)** DT-109-mediated inhibitory effect on the osteogenic differentiation-related genes *SRY-Box transcription factor 9* (*SOX9*), *bone morphogenetic protein 2 (BMP2*), and *matrix metallopeptidase 11 (MMP11*) was not obvious as detected by real-time PCR (n=5). **(e)** After 24 h ox-LDL induction, the protein level of runx family transcription factor 2 (RUNX2) was detected by western blot in DT-109-treated human aortic smooth muscle cells (n=4). Data are represented as mean ± SD. *Kruskal–Wallis* test was applied for two group comparisons. Statistical differences among multiple groups were compared using one-way ANOVA with *Tukey’s post hoc* analysis or *Dunn’s* test, multiple comparison adjusted *p*-values were reported.

**
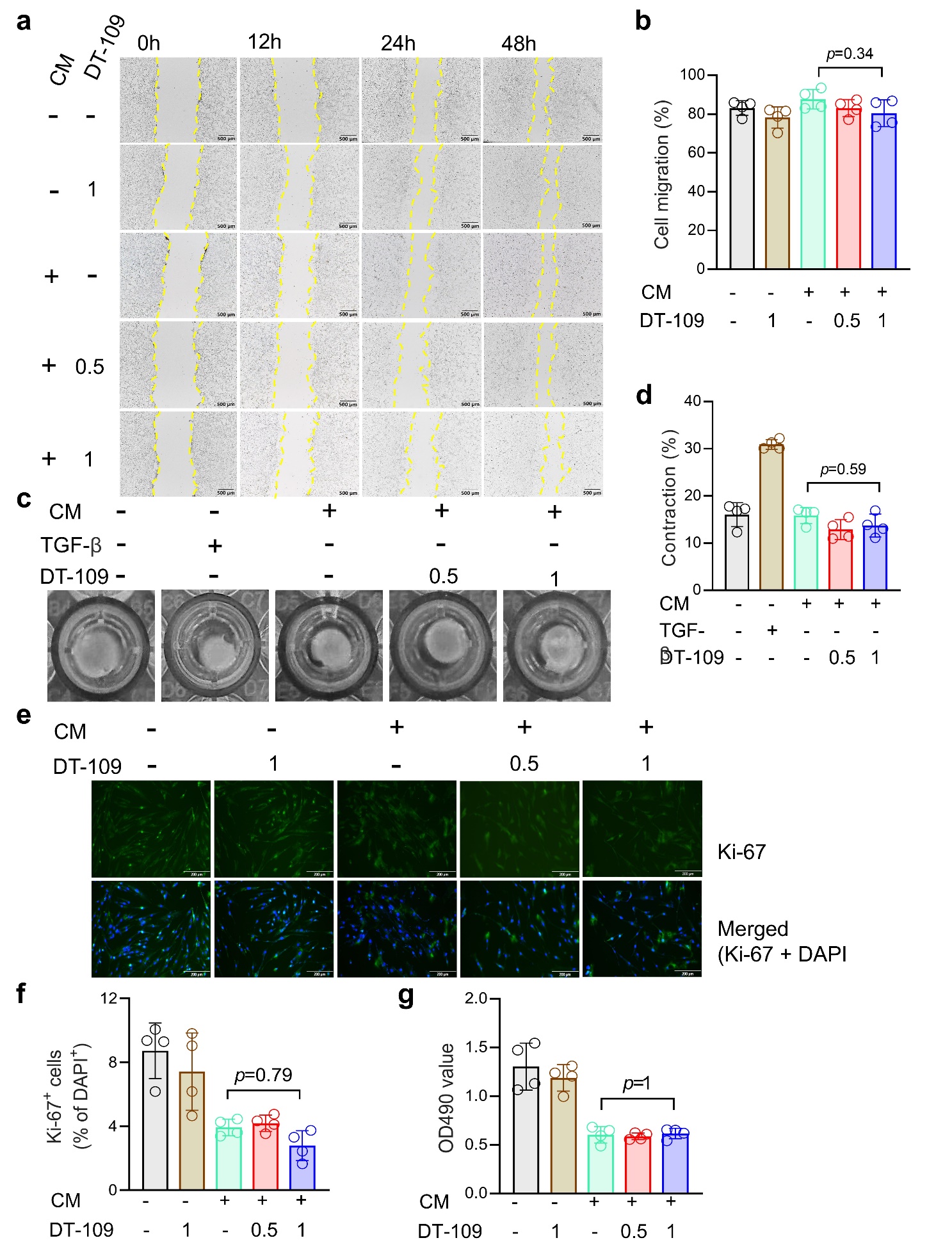
**

**Supplementary Fig. 10 DT-109 has no significant effect on SMC migration, contraction, and proliferation in response to calcifying medium.**

**(a-b)** Cells were induced with calcifying medium for 2 days, while the administered group was treated with DT-109. The scratch assay was performed to detect and quantify the migration of cells for 2 days (n=4). **(c-d)** HASMC cells were induced with calcified medium for 2 days, while the administered group was treated with DT-109, and a TGF-β positive control group was set up. A collagen gel contraction assay was performed to evaluate the cell construction. The percentage of the contracted area was quantified (n=4). **(e-f)** HASMC cells were induced with calcifying medium for 2 days while the administered group was treated with DT-109. Then Ki-67 immunofluorescence staining was used to identify the proliferating cells. The positive ratio over DAPI staining was calculated (n=4). **(g)** HASMC cells were cultured in a calcifying medium for 2 days. The concurrently administered group was treated with DT-109. Then CCK-8 was treated for 30 min and absorbance assay was performed (n=4). Data are represented as mean ± SD. Statistical differences among multiple groups were compared using one-way ANOVA with *Tukey’s post hoc* analysis or *Dunn’s* test, multiple comparison adjusted *p*-values were reported.

**
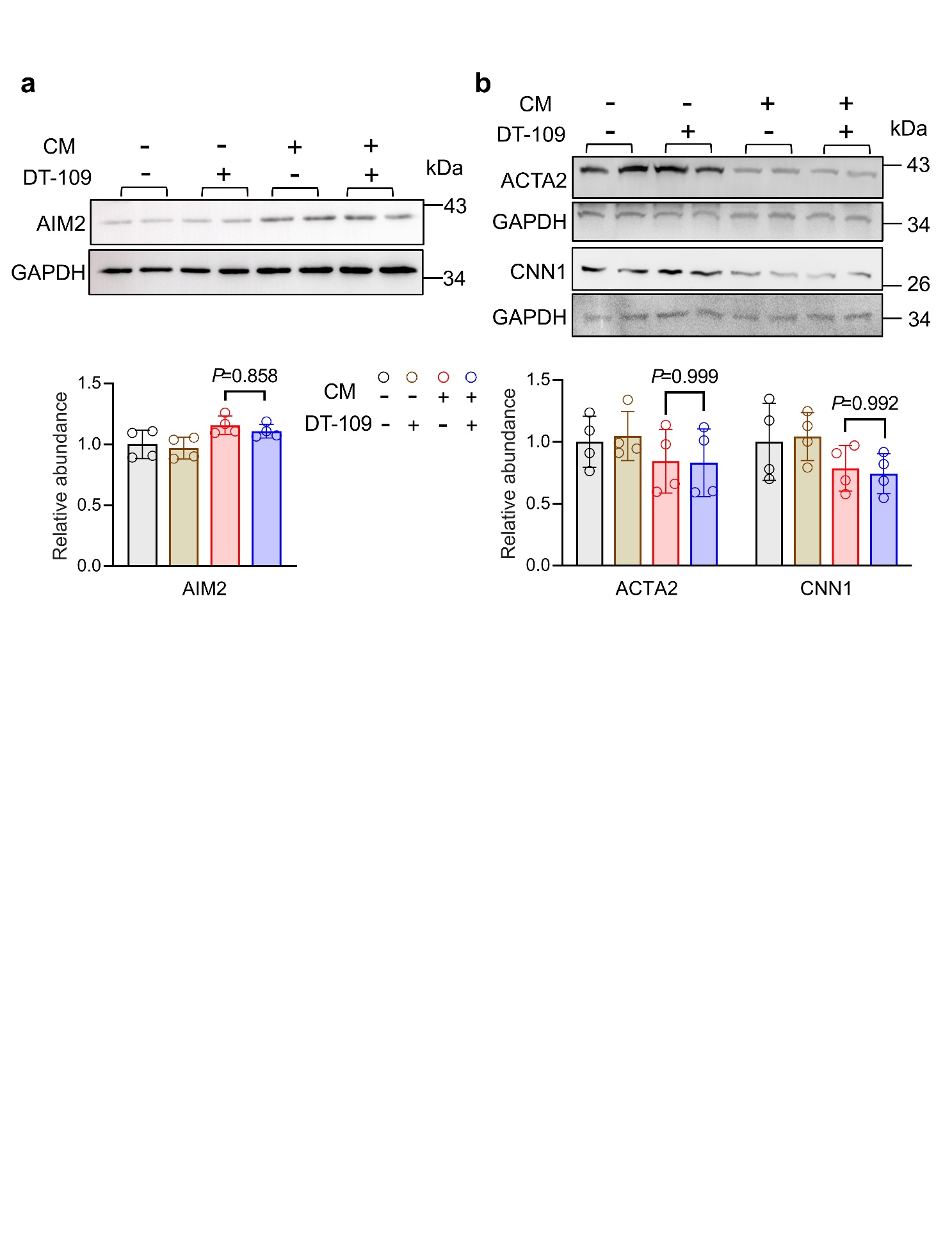
**

**Supplementary Fig. 11 Effect of DT-109 on calcifying medium-induced AIM2, ACTA2, and CNN1 expression in smooth muscle cells.**

**(a)** Protein abundance of AIM2 (absent in melanoma 2) in DT-109-treated A7r5 cells was detected by Western blot after 5 days of calcifying medium (CM) induction (n=4). **(b)** The protein levels of ACTA2 (actin alpha 2, smooth muscle) and CNN1 (calponin 1) in DT-109-treated A7r5 cells were detected by western blot under CM induction for 7 days (n=4). Data are represented as mean ± SD. Statistical differences among multiple groups were compared using one-way ANOVA with *Tukey’s post hoc* analysis or *Dunn’s* test, multiple comparison adjusted *p*-values were reported.

**
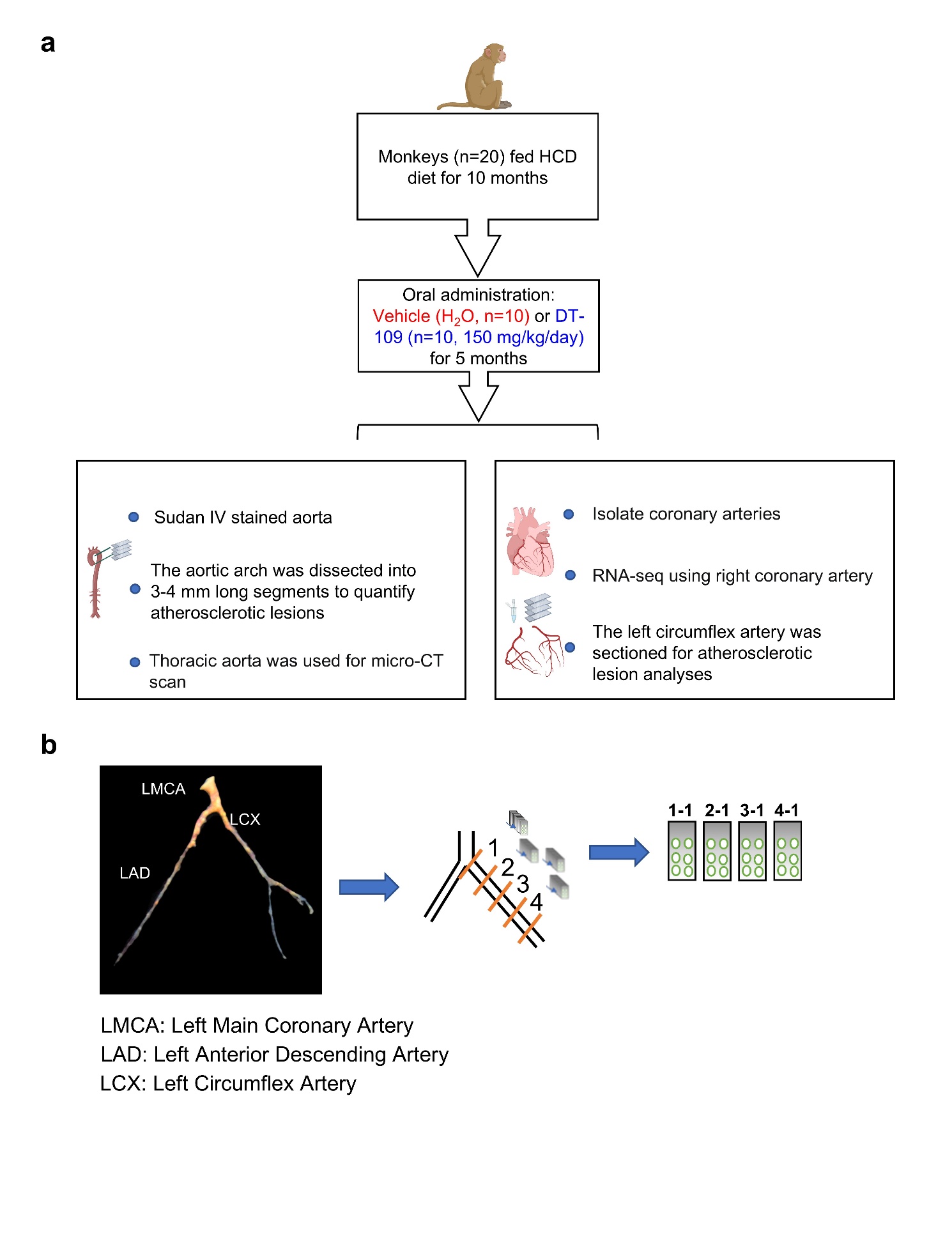
**

**Supplementary Fig. 12 Experimental protocols and methods of pathological analysis of monkey aorta and coronary artery.**

**(a)** Schematic diagram of the experimental design. Twenty male monkeys were selected to evaluate the atherosclerotic lesion of the aorta, pathological analysis of the aortic arch, and RNA-seq transcriptomics analysis of the right coronary arteries, upon receiving an HCD for 10 months, followed by DT-109 treatment for 5 months (vehicle, n=10; DT-109, n=10). **(b)** Schematic diagram of a slice of the left circumflex (LCX) of the coronary artery. The LCX was cut into four segments (approx. 500 µm in length, labeled 1-4 as in the figure). 10 serial sections of each segment were sectioned (5 µm in thickness) and used for pathological analysis and comparisons. (n=6 for each group).

**Table 1. The PCR primers used in the study.**

| Genes | Primers Forward | Primers Reverse |
| --- | --- | --- |
| 18S | CGGACATCTAAGGGCATCACAG | GGTGGAGCGATTTGTCTGGTTA |
| RUNX2 | CATGTCCCTCGGTATGTCCG | ACTCTGGCTTTGGGAAGAGC |
| SOX9 | TTCACCTACATGAACCCCGC | AGCTGTGTGTAGACGGGTTG |
| POR | CTATGGGATGCGAGGCATGT | AGAAGTCCTGGGCATTGTCG |
| BMP2 | ATTCGTGGTGGAAGTGACCC | ATCAGCCAGAGGAAAAGGGC |
| NLRP3 | TGCATGCCGTATCTGGTTGT | ACGGCGTTAGCAGAAATCCA |
| CASP1 | AACCATGGTGTGGTGTGGTT | CCTGCCCACAGACATTCGTA |
| IL-18 | CTACCAGCAAACATCTCACTTCAG | CAACTGAGAGGCTGTGCCCT |
| IL-1β | AGCTTCAGGAAGGCAGTGTC | TCAGACAGCACGAGGCATTT |
| ACTA2 | ACCATCGGGAATGAACGCTT | CTGTCAGCAATGCCTGGGTA |
| CNN1 | CGAGCCGGAGAAGCTAAGAG | TTGGACCCCGTAGCACTAGA |
| MMP2 | GGTGGCAATGGAGATGGACA | CCGGTCATAATCCTCGGTGG |
| MMP9 | GATCCCCAGAGCGTTACTCG | GTTGTGGAAACTCACACGCC |
| MMP11 | CACCGGAGAATCACCGTCAC | GCACCTGCTCCCTTACAAGT |
| NCF2 | ACTTCCAACGAGGGATGCTC | GGCTCAGACTTCATGCTCGT |
| NCF4 | CAAAAGCGTGTCCCCACAAG | CGATGTCTTTGACGGTGCTG |
| ACSL4 | TGGTTCTACTGGCCGACCTA | GTCCTTTGGTCCCAGTCCAG |
| ALFM2 | AGACGGACAAAGGCACAGAG | ATGAGCCGGCCCACATAAAA |
| TAGLN | TGGCGTGATTCTGAGCAAGT | GTTCTTGGTCACGGCCAAAC |

**Table 2. Antibodies and reagents used in the study**

Antibodies

| Reagent | Source | Identifier |
| --- | --- | --- |
| Anti-CD68 antibody | Sigma-Aldrich | Cat# HPA048982 |
| Goat anti-Rabbit IgG (H+L) Secondary antibody, HRP | Absin | Cat# abs20040ss |
| Alpha-SMA antibody | [R&D Systems](https://www.baidu.com/link?url=DrRZ79O3zvuNWIGCgQYm3s60LTzP_stv64R37iqwc4m3ikBQh0CEjg6kDS38BVGT&wd=&eqid=ca4642680001dd580000000365407d09) | MAB 1420 |
| Rabbit Anti-NALP3/CIAS1 antibody | Bioss Antibodies | bs-10021R |
| AIM2 antibody | Proteintech | 66902-1-lg |
| Rabbit Anti-ASC antibody | Bioss Antibodies | bs-6741R |
| Caspase-1 antibody | Novus Biologicals | NBP1-45433 |
| IL-1 beta/IL-1F2 antibody | Novus Biologicals | NB600-633 |
| GAPDH antibody | Proteintech | 10494-1-AP |
| Calponin 1(CNN1) antibody | Cell Signaling Technology | #17819s |
| RUNX2 antibody | Proteintech | 20700-1-AP |
| COL1A1 antibody | Cell Signaling Technology | #72026 |
| MMP2 antibody | Beyotime | AF1420 |
| MMP9 antibody | Beyotime | AF5234 |
| NOXA2/p67phox | Proteintech | 15551-1AP |

Chemicals, Peptides, and Recombinant Proteins

| Hematoxylin and Eosin Staining Kit | Beyotime | Cat# C0105S |
| --- | --- | --- |
| Von Kossa | Solarbio | Cat# 7783-99-5 |
| Sudan IV | Solarbio | Cat# S5770 |
| Ketamine hydrochloride | Zhongmu Beikang | Cat# N/A |
| Phosphate Buffered Saline | Absin | Cat# abs971 |
| OCT Compound | Sakura Tissue-Tek | Cat# 4583 |
| EDTA buffer | Maxim | Cat# 0099 |
| Hydrogen peroxide | Damao | Cat# 522 |
| Goat serum | Absin | Cat# abs933 |
| Q5 High-Fidelity DNA Polymerase | NEB | Cat# M0491 |
| Trizol reagent | Invitrogen | Cat# 15596018 |
| Ammonium acetate, LC-MS grade | MERCK | Cat# 73594 |
| Methanol, LC-MS Grade | Fischer Scientific | Cat# A456-500 |
| Water, MS-grade | Fischer Scientific | Cat# 7732-18-5 |
| Ca^2+^ Detection Assay | Nanjing Jiancheng | Cat# A070-2-4 |
| MDA Detection Assay | Nanjing Jiancheng | Cat# A003-1-1 |
| GSH Detection Assay | Nanjing Jiancheng | Cat# A006-2-1 |
| 3, 3-diaminobenzidine (DAB) kit | ZSGB-BIO | Cat# ZLI9018 |
| TransScript One-Step gDNA Removal and cDNA Synthesis SuperMix | TransGen Biotech | Cat# AT311 |
| PerfectStart Green qPCR SuperMix (+Dye II) | TransGen Biotech | Cat# AQ602 |
| DMEM with high glucose | Hyclone | Cat# SH30022.01 |
| DMEM/F-12 | Sigma | Cat# D6421 |
| FBS | ThermoFisher | Cat# 10099158 |
| 100 units per mL penicillin, and 100 μg mL−1 streptomycin | HyClone | Cat# SV30010 |
| B.C.A. protein assay kit | ZHHC | CAT# PQ003 |
| A7r5 cell | ATCC | CAT# AC339542 |
| HASMC cell | ATCC | CAT# AC339826 |
| MOVAS cell line | ATCC | CAT# AC338213 |

**Table 3. Non-standard abbreviations and acronyms**

| SMC | Smooth muscle cells |
| --- | --- |
| NLRP3 | NLR family pyrin domain containing 3 |
| AIM2 | Absent in melanoma 2 |
| CASP1 | Caspase 1 |
| NCF2 | Neutrophil cytosolic factor 2 |
| NCF4 | Neutrophil cytosolic factor 4 |
| RUNX2 | Runx family transcription factor 2 |
| COL1A1 | Collagen type I alpha 1 chain |
| MMP2 | Matrix metallopeptidase 2 |
| MMP9 | Matrix metallopeptidase 9 |
| ACTA2 | Actin alpha 2, smooth muscle |
| CNN1 | Calponin 1 |
| TAGLN | Transgelin |
| NASH | Nonalcoholic steatohepatitis |
| LPS | Lipopolysaccharides |
| NAS | NAFLD activity score |
| CA | Coronary atherosclerosis |
| HCD | High cholesterol diet |
| SOX9 | SRY-Box transcription factor 9 |
| POR | Cytochrome p450 oxidoreductase |
| BMP2 | Bone morphogenetic protein 2 |
| ASC (PYCARD) | PYD and CARD domain containing |
| IL-18 | Interleukin 18 |
| IL-1β | Interleukin 1 beta |
| ACSL4 | Acyl-CoA synthetase long chain family member 4 |
| GSH | Glutathione |
| MDA | Malondialdehyde |
| ox-LDL | Oxidized low-density lipoprotein |
| MYH11 | Myosin heavy chain 11 |
| MYLK | Myosin light chain kinase |
| ROS | Reactiveoxygenspecies |
| HCD | High-cholesterol diet |
| TC | Total cholesterol |
| TG | Triglyceride |
| LDL-c | Low-density lipoprotein cholesterol |

**Western blot results and the uncropped images.**
